# 6,12,18,24-Tetra­meth­oxy-4,10,16,22-tetra­kis­[(meth­oxy­carbon­yl)meth­oxy]-2,8,14,20-tetra­kis­(2-phenyl­eth­yl)resorcin[4]arene

**DOI:** 10.1107/S1600536811051567

**Published:** 2011-12-10

**Authors:** Pramod B. Pansuriya, Holger B. Friedrich, Glenn E. M. Maguire

**Affiliations:** a School of Chemistry, University of KwaZulu-Natal, Durban 4000, South Africa

## Abstract

The title compound, C_76_H_80_O_16_, is a macrocyclic structure. This novel resorcin[4]arene derivative has (meth­oxy­carbon­yl)meth­oxy ‘head’ groups on the upper rim. The compound has a *C*_2*v*_ ‘boat’ geometry and there are a range of C—H⋯O contacts in the crystal structure.

## Related literature

For applications of resorcin[4]arenes, see: Ajami *et al.* (2011 ▶); Sun *et al.* (2010 ▶); Arnott *et al.* (2006 ▶); Sokoliess *et al.* (2002 ▶). For structural information, see: Wiegmann & Mattay (2011 ▶); Pansuriya *et al.* (2011 ▶). For details of C—H⋯π inter­actions, see: Nishio (2004 ▶). For the synthesis of tetra­meth­oxy resorcin[4]arenes: Mclldowie *et al.* (2000 ▶).
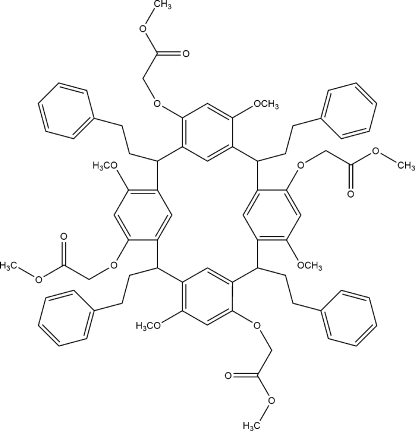


## Experimental

### 

#### Crystal data

C_76_H_80_O_16_
*M* *_r_* = 1249.40
Monoclinic, 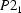

*a* = 14.1361 (7) Å
*b* = 32.2264 (17) Å
*c* = 28.9417 (16) Å
β = 90.572 (1)°
*V* = 13183.9 (12) Å^3^
*Z* = 8
Mo *K*α radiation
μ = 0.09 mm^−1^
*T* = 173 K
0.41 × 0.33 × 0.29 mm

#### Data collection

Bruker Kappa DUO APEXII diffractometer
Absorption correction: multi-scan (*SADABS*; Bruker, 2006 ▶) *T*_min_ = 0.965, *T*_max_ = 0.975
126671 measured reflections
33391 independent reflections
23289 reflections with *I* > 2σ(*I*)
*R*_int_ = 0.072

#### Refinement

*R*[*F* ^2^ > 2σ(*F* ^2^)] = 0.056
*wR*(*F* ^2^) = 0.162
*S* = 1.02
33391 reflections
3345 parameters
1 restraint
H-atom parameters constrained
Δρ_max_ = 0.44 e Å^−3^
Δρ_min_ = −0.33 e Å^−3^

### 

Data collection: *APEX2* (Bruker, 2006 ▶); cell refinement: *SAINT* (Bruker, 2006 ▶); data reduction: *SAINT*; program(s) used to solve structure: *SHELXS97* (Sheldrick, 2008 ▶); program(s) used to refine structure: *SHELXL97* (Sheldrick, 2008 ▶); molecular graphics: *OLEX2* (Dolomanov *et al.*, 2009 ▶); software used to prepare material for publication: *SHELXL97*.

## Supplementary Material

Crystal structure: contains datablock(s) I, global. DOI: 10.1107/S1600536811051567/hg5129sup1.cif

Structure factors: contains datablock(s) I. DOI: 10.1107/S1600536811051567/hg5129Isup2.hkl

Additional supplementary materials:  crystallographic information; 3D view; checkCIF report

## Figures and Tables

**Table 1:** Hydrogen-bond geometry (Å, °)

*D*—H⋯*A*	*D*—H	H⋯*A*	*D*⋯*A*	*D*—H⋯*A*
C14*B*—H14*B*⋯O16*A*^i^	1.00	2.59	3.440 (4)	142
C30*B*—H30*C*⋯O11*A*^i^	0.99	2.49	3.464 (6)	169
C30*C*—H30*F*⋯O8*D*^ii^	0.99	2.55	3.259 (4)	128
C30*D*—H30*G*⋯O8*C*^iii^	0.99	2.56	3.270 (5)	129
C44*A*—H44*B*⋯O10*B*^iv^	0.98	2.54	3.292 (5)	133
C56*C*—H56*H*⋯O7*B*^v^	0.98	2.55	3.375 (9)	141
C56*D*—H56*K*⋯O15*A*^vi^	0.98	2.37	3.251 (8)	149
C62*C*—H62*C*⋯O7*C*^vii^	0.95	2.46	3.313 (6)	149
C62*D*—H62*D*⋯O7*D*^vii^	0.95	2.59	3.421 (7)	146
C63*A*—H63*A*⋯O3*B*^viii^	0.95	2.37	3.267 (7)	156

Symmetry codes: (i) 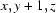; (ii) 
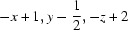
; (iii) 
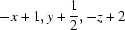
; (iv) 
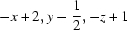
; (v) 
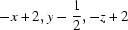
; (vi) 
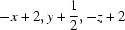
; (vii) 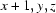; (viii) 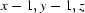.

**Fig. 1.**
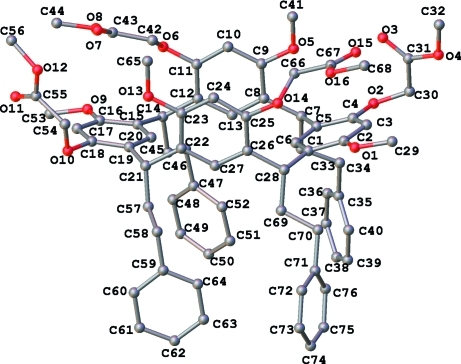
The molecular structure of the title compound with atomic numbering; the H atoms have been omitted for clarity.

**Fig. 2.**
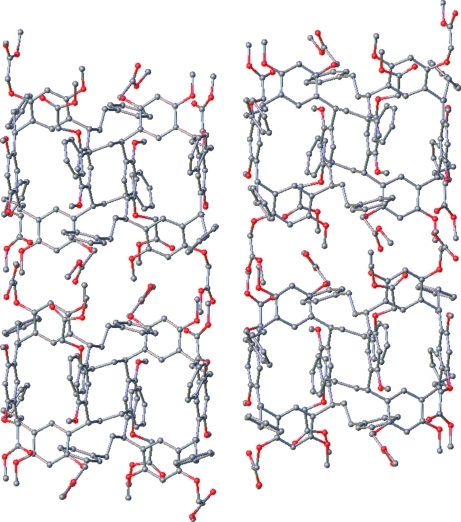
The packing of the title compound in [100] plane. All hydrogen atoms have been omitted for clarity.

## supplementary crystallographic information

### Comment

Resorcin[4]arene derivatives with different physicochemical properties have received extensive research interest (Ajami *et al.*, 2011; Sun *et al.*, 2010; Arnott *et al.*, 2006; Sokoliess *et al.*, 2002). One such family of relevant molecules that has been reported on is the semi-flexible tetramethoxy resorcin[4]arenes (Wiegmann & Mattay 2011). Here we report the crystal structure of a novel tetramethoxy resorcin[4]arene derivative containing (methoxycarbonyl)methoxy "head" groups on the upper rim.

The title compound is racemic and has an *rccc* (boat) configuration (Fig. 1). This differs from our previuosly reported octamethoxy resorcin[4]arene structure which had a chair (*rctt*) configuration (Pansuriya *et al.*, 2011). Several non-classical inter- and intramolecular weak hydrogen bonds are present in the structure. C— H···π contacts (Nishio, 2004) involving methoxy, ester groups and neighbouring aromatic rings with H···π distances (centroid of the aromatic ring) ranging from 2.62 to 2.96Å are present. Interlocking of (methoxycarbonyl)methoxy head groups on the upper rim and interdigitation of the "feet" in lower rim create infinite colums these interactions in [100] plane (Fig. 2).

### Experimental

In dry degased acetonitrile (100 ml) tetramethoxy resorcin[4]arene (0.58 g, 0.6 mmol) and anhydrous potassium carbonate (0.785 g, 5.7 mmol) were added. The reaction was stirred at 70 °C for ten minutes and then methyl acetyl bromide (425 µl, 4.6 mmol) was added dropwise. The reaction solution was allowed to reflux for 24 h and then cooled to room temperature. The solvent was reduced under vacuum and the resulting residue extracted with DCM (100 ml). The DCM was washed with 1*M* HCl (50 ml), water (50 ml) and then brine (50 ml). The organic layer was separated and dried over anhydrous magnesium sulfate. The solvent was reduced to complete dryness to afford the product as a white solid (Yield: 0.60 g, 80%), *M*.p. 387 K.

Crystal suitable for X-ray diffraction analysis were grown at room temperature from a solution of DCM:methanol (2:1).

### Refinement

All non-hydrogen atoms were refined anisotropically. All hydrogen atoms were placed at calculated positions with attach distances ranging from 0.95Å to 1.00Å and refined as riding on their parent atoms with *U*_iso_ (H) = 1.2 or 1.5 *U*_eq_ (C).

### Crystal data

**Table:**

C_76_H_80_O_16_	PHTMTMES
*M**_r_* = 1249.40	*D*_x_ = 1.259 Mg m^−^^3^
Monoclinic, *P*2_1_	Melting point: 387 K
Hall symbol: P 2yb	Mo *K*α radiation, λ = 0.71073 Å
*a* = 14.1361 (7) Å	Cell parameters from 126694 reflections
*b* = 32.2264 (17) Å	θ = 1.6–28.3°
*c* = 28.9417 (16) Å	µ = 0.09 mm^−^^1^
β = 90.572 (1)°	*T* = 173 K
*V* = 13183.9 (12) Å^3^	Block, colourless
*Z* = 8	0.41 × 0.33 × 0.29 mm
*F*(000) = 5312	

### Data collection

**Table:**

Bruker Kappa DUO APEXII diffractometer	33391 independent reflections
Radiation source: fine-focus sealed tube	23289 reflections with *I* > 2σ(*I*)
graphite	*R*_int_ = 0.072
0.5° φ scans and ω scans	θ_max_ = 28.3°, θ_min_ = 1.6°
Absorption correction: multi-scan (*SADABS*; Bruker, 2006)	*h* = −18→18
*T*_min_ = 0.965, *T*_max_ = 0.975	*k* = −42→42
126671 measured reflections	*l* = −38→38

### Refinement

**Table:**

Refinement on *F*^2^	Primary atom site location: structure-invariant direct methods
Least-squares matrix: full	Secondary atom site location: difference Fourier map
*R*[*F*^2^ > 2σ(*F*^2^)] = 0.056	Hydrogen site location: inferred from neighbouring sites
*wR*(*F*^2^) = 0.162	H-atom parameters constrained
*S* = 1.02	*w* = 1/[σ^2^(*F*_o_^2^) + (0.0912*P*)^2^ + 0.241*P*] where *P* = (*F*_o_^2^ + 2*F*_c_^2^)/3
33391 reflections	(Δ/σ)_max_ = 0.001
3345 parameters	Δρ_max_ = 0.44 e Å^−^^3^
1 restraint	Δρ_min_ = −0.33 e Å^−^^3^

### Special details

**Table:**

Geometry. All e.s.d.'s (except the e.s.d. in the dihedral angle between two l.s. planes) are estimated using the full covariance matrix. The cell e.s.d.'s are taken into account individually in the estimation of e.s.d.'s in distances, angles and torsion angles; correlations between e.s.d.'s in cell parameters are only used when they are defined by crystal symmetry. An approximate (isotropic) treatment of cell e.s.d.'s is used for estimating e.s.d.'s involving l.s. planes.
Refinement. Refinement of *F*^2^ against ALL reflections. The weighted *R*-factor *wR* and goodness of fit *S* are based on *F*^2^, conventional *R*-factors *R* are based on *F*, with *F* set to zero for negative *F*^2^. The threshold expression of *F*^2^ > σ(*F*^2^) is used only for calculating *R*-factors(gt) *etc*. and is not relevant to the choice of reflections for refinement. *R*-factors based on *F*^2^ are statistically about twice as large as those based on *F*, and *R*- factors based on ALL data will be even larger.

### Fractional atomic coordinates and isotropic or equivalent isotropic displacement parameters (Å^2^)

**Table:**

	*x*	*y*	*z*	*U*_iso_*/*U*_eq_	
O1A	0.5977 (2)	0.06377 (9)	0.62469 (8)	0.0406 (7)	
O2A	0.73848 (19)	0.19974 (8)	0.63413 (8)	0.0345 (6)	
O3A	0.8421 (3)	0.15922 (14)	0.70321 (12)	0.0741 (11)	
O4A	0.9771 (2)	0.17877 (12)	0.66923 (14)	0.0676 (10)	
O5A	0.90039 (17)	0.18472 (9)	0.52995 (8)	0.0353 (6)	
O6A	0.91179 (17)	0.19634 (8)	0.36350 (8)	0.0311 (5)	
O7A	0.9798 (2)	0.13462 (11)	0.27089 (11)	0.0570 (8)	
O8A	1.0272 (2)	0.19763 (9)	0.29379 (9)	0.0448 (7)	
O9A	0.7815 (2)	0.20481 (9)	0.26602 (8)	0.0383 (6)	
O10A	0.6606 (2)	0.06387 (9)	0.25759 (8)	0.0410 (7)	
O11A	0.8362 (3)	0.03043 (16)	0.23211 (16)	0.0933 (15)	
O12A	0.8293 (2)	0.07451 (13)	0.17483 (12)	0.0655 (10)	
O13A	0.79804 (19)	0.04378 (9)	0.36270 (9)	0.0410 (6)	
O14A	0.77605 (17)	0.04228 (8)	0.53017 (8)	0.0305 (5)	
O15A	0.9510 (3)	0.08568 (16)	0.60233 (14)	0.0932 (16)	
O16A	0.8347 (2)	0.04065 (9)	0.61531 (9)	0.0447 (7)	
C1A	0.6302 (2)	0.10053 (10)	0.55664 (11)	0.0249 (7)	
C2A	0.6351 (3)	0.09882 (11)	0.60507 (11)	0.0291 (8)	
C3A	0.6740 (3)	0.13123 (12)	0.62974 (11)	0.0319 (8)	
H3A	0.6800	0.1293	0.6624	0.038*	
C4A	0.7045 (3)	0.16686 (11)	0.60716 (11)	0.0281 (7)	
C5A	0.6969 (2)	0.17097 (11)	0.55929 (11)	0.0244 (7)	
C6A	0.6609 (2)	0.13684 (10)	0.53538 (10)	0.0241 (7)	
H6A	0.6570	0.1384	0.5026	0.029*	
C7A	0.7166 (2)	0.21170 (10)	0.53441 (10)	0.0235 (7)	
H7A	0.7597	0.2288	0.5544	0.028*	
C8A	0.7647 (2)	0.20475 (10)	0.48799 (11)	0.0240 (7)	
C9A	0.8593 (2)	0.19183 (11)	0.48741 (11)	0.0250 (7)	
C10A	0.9078 (2)	0.18738 (12)	0.44612 (12)	0.0289 (7)	
H10A	0.9719	0.1785	0.4463	0.035*	
C11A	0.8616 (2)	0.19601 (10)	0.40468 (11)	0.0246 (7)	
C12A	0.7668 (2)	0.20724 (10)	0.40281 (11)	0.0248 (7)	
C13A	0.7203 (2)	0.21126 (10)	0.44541 (10)	0.0243 (7)	
H13A	0.6554	0.2188	0.4451	0.029*	
C14A	0.7196 (2)	0.21474 (11)	0.35646 (11)	0.0261 (7)	
H14A	0.7629	0.2329	0.3382	0.031*	
C15A	0.7084 (2)	0.17450 (11)	0.33004 (10)	0.0251 (7)	
C16A	0.7378 (2)	0.17095 (12)	0.28416 (11)	0.0282 (7)	
C17A	0.7240 (3)	0.13437 (12)	0.25938 (11)	0.0308 (8)	
H17A	0.7439	0.1325	0.2282	0.037*	
C18A	0.6811 (3)	0.10060 (12)	0.28029 (11)	0.0311 (8)	
C19A	0.6537 (2)	0.10221 (11)	0.32679 (11)	0.0278 (7)	
C20A	0.6681 (2)	0.13929 (11)	0.34998 (11)	0.0268 (7)	
H20A	0.6494	0.1409	0.3814	0.032*	
C21A	0.6119 (3)	0.06472 (12)	0.35088 (11)	0.0295 (8)	
H21A	0.6293	0.0396	0.3325	0.035*	
C22A	0.6556 (3)	0.05972 (10)	0.39928 (11)	0.0271 (7)	
C23A	0.7510 (3)	0.04899 (11)	0.40365 (12)	0.0305 (7)	
C24A	0.7927 (2)	0.04358 (11)	0.44714 (12)	0.0296 (7)	
H24A	0.8578	0.0367	0.4498	0.035*	
C25A	0.7386 (2)	0.04828 (11)	0.48638 (11)	0.0261 (7)	
C26A	0.6427 (2)	0.05874 (10)	0.48386 (11)	0.0242 (7)	
C27A	0.6043 (2)	0.06467 (10)	0.43976 (11)	0.0250 (7)	
H27A	0.5397	0.0725	0.4372	0.030*	
C28A	0.5875 (2)	0.06508 (11)	0.52819 (11)	0.0251 (7)	
H28A	0.5938	0.0391	0.5469	0.030*	
C29A	0.5862 (5)	0.06332 (18)	0.67372 (14)	0.0703 (17)	
H29A	0.6481	0.0662	0.6889	0.106*	
H29B	0.5572	0.0370	0.6830	0.106*	
H29C	0.5454	0.0864	0.6829	0.106*	
C30A	0.8390 (3)	0.20284 (14)	0.63534 (13)	0.0378 (9)	
H30A	0.8572	0.2323	0.6396	0.045*	
H30B	0.8644	0.1935	0.6053	0.045*	
C31A	0.8827 (3)	0.17742 (15)	0.67339 (15)	0.0477 (11)	
C32A	1.0308 (5)	0.1546 (2)	0.7027 (3)	0.101 (2)	
H32A	1.0231	0.1666	0.7336	0.152*	
H32B	1.0979	0.1550	0.6945	0.152*	
H32C	1.0078	0.1259	0.7027	0.152*	
C33A	0.6202 (2)	0.23448 (11)	0.53008 (12)	0.0277 (7)	
H33A	0.5799	0.2190	0.5078	0.033*	
H33B	0.5887	0.2334	0.5605	0.033*	
C34A	0.6245 (3)	0.27998 (11)	0.51453 (12)	0.0305 (7)	
H34A	0.6579	0.2966	0.5383	0.037*	
H34B	0.6605	0.2820	0.4855	0.037*	
C35A	0.5263 (2)	0.29722 (11)	0.50698 (12)	0.0288 (7)	
C36A	0.4719 (3)	0.31067 (13)	0.54413 (13)	0.0377 (9)	
H36A	0.4983	0.3102	0.5745	0.045*	
C37A	0.3806 (3)	0.32462 (14)	0.53750 (15)	0.0453 (10)	
H37A	0.3444	0.3333	0.5632	0.054*	
C38A	0.3416 (3)	0.32607 (14)	0.49362 (16)	0.0447 (10)	
H38A	0.2790	0.3360	0.4890	0.054*	
C39A	0.3938 (3)	0.31306 (15)	0.45668 (15)	0.0445 (10)	
H39A	0.3669	0.3138	0.4265	0.053*	
C40A	0.4859 (3)	0.29885 (14)	0.46317 (13)	0.0399 (9)	
H40A	0.5215	0.2901	0.4373	0.048*	
C41A	0.9945 (3)	0.16995 (15)	0.53127 (14)	0.0433 (10)	
H41A	0.9982	0.1437	0.5143	0.065*	
H41B	1.0141	0.1655	0.5634	0.065*	
H41C	1.0362	0.1904	0.5169	0.065*	
C42A	0.9395 (3)	0.15703 (12)	0.34657 (12)	0.0333 (8)	
H42A	0.8836	0.1386	0.3443	0.040*	
H42B	0.9857	0.1442	0.3682	0.040*	
C43A	0.9834 (3)	0.16199 (13)	0.29944 (13)	0.0379 (9)	
C44A	1.0727 (4)	0.20459 (17)	0.25007 (16)	0.0601 (13)	
H44A	1.1317	0.1887	0.2490	0.090*	
H44B	1.0867	0.2342	0.2466	0.090*	
H44C	1.0306	0.1956	0.2249	0.090*	
C45A	0.6238 (3)	0.23760 (11)	0.35982 (11)	0.0290 (7)	
H45A	0.5770	0.2191	0.3745	0.035*	
H45B	0.6315	0.2624	0.3796	0.035*	
C46A	0.5866 (3)	0.25087 (16)	0.31208 (13)	0.0473 (11)	
H46A	0.6371	0.2659	0.2956	0.057*	
H46B	0.5702	0.2258	0.2939	0.057*	
C47A	0.5011 (3)	0.27824 (15)	0.31537 (12)	0.0425 (10)	
C48A	0.4112 (3)	0.26474 (17)	0.30256 (13)	0.0494 (11)	
H48A	0.4024	0.2370	0.2924	0.059*	
C49A	0.3340 (3)	0.2913 (2)	0.30441 (15)	0.0573 (14)	
H49A	0.2733	0.2817	0.2950	0.069*	
C50A	0.3447 (4)	0.3311 (2)	0.31966 (18)	0.0648 (15)	
H50A	0.2914	0.3490	0.3212	0.078*	
C51A	0.4325 (4)	0.34521 (18)	0.33273 (19)	0.0665 (14)	
H51A	0.4404	0.3729	0.3433	0.080*	
C52A	0.5091 (3)	0.31913 (17)	0.33049 (16)	0.0536 (12)	
H52A	0.5695	0.3293	0.3396	0.064*	
C53A	0.8011 (4)	0.20537 (17)	0.21830 (13)	0.0577 (13)	
H53A	0.8451	0.1828	0.2110	0.087*	
H53B	0.8298	0.2320	0.2101	0.087*	
H53C	0.7422	0.2016	0.2007	0.087*	
C54A	0.6866 (3)	0.05979 (14)	0.21032 (13)	0.0407 (9)	
H54A	0.6692	0.0854	0.1933	0.049*	
H54B	0.6519	0.0363	0.1961	0.049*	
C55A	0.7913 (3)	0.05247 (14)	0.20686 (15)	0.0471 (11)	
C56A	0.9316 (4)	0.0719 (2)	0.1711 (2)	0.0755 (16)	
H56A	0.9596	0.0677	0.2019	0.113*	
H56B	0.9560	0.0976	0.1577	0.113*	
H56C	0.9482	0.0484	0.1512	0.113*	
C57A	0.5038 (3)	0.06717 (13)	0.35165 (11)	0.0325 (8)	
H57A	0.4849	0.0932	0.3672	0.039*	
H57B	0.4792	0.0437	0.3701	0.039*	
C58A	0.4580 (3)	0.06585 (15)	0.30347 (12)	0.0419 (10)	
H58A	0.4818	0.0894	0.2850	0.050*	
H58B	0.4767	0.0399	0.2878	0.050*	
C59A	0.3524 (3)	0.06807 (14)	0.30531 (12)	0.0379 (9)	
C60A	0.3049 (3)	0.10405 (16)	0.29462 (14)	0.0479 (11)	
H60A	0.3398	0.1275	0.2844	0.057*	
C61A	0.2061 (3)	0.1071 (2)	0.29837 (17)	0.0613 (13)	
H61A	0.1749	0.1325	0.2915	0.074*	
C62A	0.1555 (4)	0.0736 (2)	0.31188 (16)	0.0644 (15)	
H62A	0.0886	0.0754	0.3141	0.077*	
C63A	0.2002 (4)	0.0374 (2)	0.32229 (17)	0.0691 (17)	
H63A	0.1641	0.0140	0.3313	0.083*	
C64A	0.2986 (4)	0.03417 (16)	0.31990 (15)	0.0517 (11)	
H64A	0.3291	0.0089	0.3282	0.062*	
C65A	0.8986 (3)	0.04058 (14)	0.36390 (15)	0.0449 (10)	
H65A	0.9248	0.0624	0.3839	0.067*	
H65B	0.9233	0.0438	0.3326	0.067*	
H65C	0.9169	0.0133	0.3761	0.067*	
C66A	0.8643 (3)	0.06057 (17)	0.53789 (14)	0.0505 (11)	
H66A	0.8646	0.0887	0.5240	0.061*	
H66B	0.9135	0.0439	0.5224	0.061*	
C67A	0.8881 (3)	0.06384 (16)	0.58885 (15)	0.0502 (11)	
C68A	0.8574 (5)	0.04117 (18)	0.66394 (16)	0.0662 (15)	
H68A	0.9216	0.0305	0.6688	0.099*	
H68B	0.8123	0.0237	0.6806	0.099*	
H68C	0.8538	0.0697	0.6755	0.099*	
C69A	0.4809 (2)	0.07354 (11)	0.52119 (12)	0.0296 (7)	
H69A	0.4542	0.0822	0.5512	0.035*	
H69B	0.4736	0.0972	0.4996	0.035*	
C70A	0.4216 (3)	0.03694 (12)	0.50242 (13)	0.0346 (8)	
H70A	0.4499	0.0268	0.4734	0.041*	
H70B	0.4232	0.0139	0.5251	0.041*	
C71A	0.3203 (3)	0.04940 (13)	0.49332 (13)	0.0367 (8)	
C72A	0.2949 (3)	0.06849 (15)	0.45133 (15)	0.0474 (10)	
H72A	0.3411	0.0719	0.4280	0.057*	
C73A	0.2032 (3)	0.08246 (17)	0.44343 (17)	0.0548 (12)	
H73A	0.1868	0.0950	0.4147	0.066*	
C74A	0.1361 (3)	0.07811 (18)	0.47733 (19)	0.0620 (14)	
H74A	0.0736	0.0880	0.4721	0.074*	
C75A	0.1591 (3)	0.0596 (2)	0.51867 (17)	0.0628 (15)	
H75A	0.1125	0.0563	0.5418	0.075*	
C76A	0.2520 (3)	0.04549 (18)	0.52660 (15)	0.0512 (11)	
H76A	0.2679	0.0331	0.5554	0.061*	
O1B	1.0018 (2)	0.79524 (9)	0.34133 (8)	0.0381 (7)	
O2B	0.8747 (2)	0.93555 (9)	0.33391 (8)	0.0411 (7)	
O3B	1.0602 (2)	0.95651 (13)	0.32072 (11)	0.0654 (10)	
O4B	1.0487 (3)	0.93523 (15)	0.24792 (11)	0.0755 (12)	
O5B	1.01302 (19)	0.95766 (10)	0.43576 (8)	0.0399 (7)	
O6B	1.00419 (19)	0.96042 (9)	0.60273 (8)	0.0378 (6)	
O7B	1.1619 (4)	0.90759 (19)	0.67830 (14)	0.1072 (18)	
O8B	1.0632 (3)	0.96019 (13)	0.68735 (11)	0.0770 (12)	
O9B	0.8300 (2)	0.93672 (9)	0.70036 (9)	0.0490 (8)	
O10B	0.96121 (18)	0.79857 (8)	0.70902 (8)	0.0323 (6)	
O11B	1.0645 (3)	0.83863 (15)	0.77772 (13)	0.0826 (13)	
O12B	1.2000 (3)	0.81789 (13)	0.74503 (16)	0.0787 (12)	
O13B	1.12398 (17)	0.81423 (9)	0.60462 (8)	0.0343 (6)	
O14B	1.13407 (18)	0.80255 (8)	0.43820 (8)	0.0334 (6)	
O15B	1.1980 (2)	0.86203 (10)	0.34318 (10)	0.0525 (8)	
O16B	1.2481 (2)	0.80046 (10)	0.36801 (9)	0.0465 (7)	
C1B	0.9267 (2)	0.82519 (11)	0.40523 (11)	0.0250 (7)	
C2B	0.9557 (2)	0.82915 (11)	0.35953 (11)	0.0278 (7)	
C3B	0.9405 (3)	0.86564 (12)	0.33482 (11)	0.0301 (7)	
H3B	0.9603	0.8679	0.3037	0.036*	
C4B	0.8957 (3)	0.89890 (11)	0.35642 (11)	0.0289 (7)	
C5B	0.8691 (2)	0.89670 (11)	0.40284 (11)	0.0271 (7)	
C6B	0.8852 (2)	0.85949 (11)	0.42566 (11)	0.0252 (7)	
H6B	0.8668	0.8574	0.4571	0.030*	
C7B	0.8277 (2)	0.93422 (11)	0.42711 (11)	0.0259 (7)	
H7B	0.8439	0.9591	0.4081	0.031*	
C8B	0.8740 (2)	0.94041 (10)	0.47453 (11)	0.0259 (7)	
C9B	0.9693 (2)	0.95270 (12)	0.47707 (11)	0.0296 (7)	
C10B	1.0125 (3)	0.95889 (12)	0.51966 (12)	0.0336 (8)	
H10B	1.0770	0.9671	0.5213	0.040*	
C11B	0.9617 (3)	0.95311 (12)	0.56002 (12)	0.0300 (8)	
C12B	0.8673 (3)	0.94091 (10)	0.55934 (11)	0.0263 (7)	
C13B	0.8265 (2)	0.93461 (10)	0.51559 (11)	0.0258 (7)	
H13B	0.7624	0.9258	0.5140	0.031*	
C14B	0.8148 (2)	0.93470 (10)	0.60427 (11)	0.0254 (7)	
H14B	0.8225	0.9606	0.6230	0.031*	
C15B	0.8580 (2)	0.89903 (11)	0.63205 (11)	0.0253 (7)	
C16B	0.8635 (3)	0.90096 (11)	0.68041 (11)	0.0314 (8)	
C17B	0.9006 (3)	0.86782 (12)	0.70526 (11)	0.0328 (8)	
H17B	0.9064	0.8695	0.7379	0.039*	
C18B	0.9294 (2)	0.83197 (11)	0.68235 (11)	0.0267 (7)	
C19B	0.9210 (2)	0.82820 (11)	0.63443 (10)	0.0242 (7)	
C20B	0.8859 (2)	0.86279 (11)	0.61066 (10)	0.0241 (7)	
H20B	0.8809	0.8613	0.5779	0.029*	
C21B	0.9388 (2)	0.78750 (10)	0.60906 (10)	0.0235 (7)	
H21B	0.9819	0.7703	0.6289	0.028*	
C22B	0.9877 (2)	0.79437 (10)	0.56311 (10)	0.0232 (6)	
C23B	1.0827 (2)	0.80747 (11)	0.56224 (11)	0.0280 (7)	
C24B	1.1303 (2)	0.81150 (11)	0.52095 (12)	0.0289 (7)	
H24B	1.1948	0.8197	0.5210	0.035*	
C25B	1.0840 (2)	0.80361 (11)	0.47957 (11)	0.0283 (7)	
C26B	0.9889 (2)	0.79228 (10)	0.47836 (10)	0.0233 (6)	
C27B	0.9430 (2)	0.78827 (10)	0.52042 (11)	0.0238 (7)	
H27B	0.8778	0.7810	0.5201	0.029*	
C28B	0.9405 (2)	0.78463 (10)	0.43182 (10)	0.0255 (7)	
H28B	0.9847	0.7672	0.4133	0.031*	
C29B	1.0240 (4)	0.79570 (16)	0.29342 (13)	0.0542 (13)	
H29D	0.9665	0.8013	0.2753	0.081*	
H29E	1.0500	0.7687	0.2845	0.081*	
H29F	1.0708	0.8174	0.2875	0.081*	
C30B	0.9087 (3)	0.94058 (14)	0.28801 (13)	0.0406 (9)	
H30C	0.8792	0.9654	0.2738	0.049*	
H30D	0.8908	0.9161	0.2692	0.049*	
C31B	1.0131 (3)	0.94533 (13)	0.28824 (14)	0.0432 (10)	
C32B	1.1493 (4)	0.9368 (3)	0.2451 (2)	0.091 (2)	
H32D	1.1708	0.9654	0.2500	0.136*	
H32E	1.1690	0.9273	0.2145	0.136*	
H32F	1.1772	0.9188	0.2689	0.136*	
C33B	0.7192 (2)	0.93216 (11)	0.42923 (12)	0.0302 (7)	
H33C	0.6958	0.9560	0.4475	0.036*	
H33D	0.7005	0.9064	0.4454	0.036*	
C34B	0.6721 (3)	0.93296 (13)	0.38107 (13)	0.0377 (9)	
H34C	0.6881	0.9593	0.3654	0.045*	
H34D	0.6972	0.9098	0.3623	0.045*	
C35B	0.5655 (3)	0.92903 (12)	0.38402 (12)	0.0339 (8)	
C36B	0.5113 (3)	0.95992 (14)	0.40262 (17)	0.0487 (11)	
H36B	0.5413	0.9844	0.4138	0.058*	
C37B	0.4139 (3)	0.95647 (15)	0.40565 (17)	0.0505 (11)	
H37B	0.3781	0.9783	0.4188	0.061*	
C38B	0.3689 (3)	0.92137 (15)	0.38954 (15)	0.0456 (10)	
H38B	0.3020	0.9189	0.3910	0.055*	
C39B	0.4224 (3)	0.88992 (15)	0.37124 (16)	0.0532 (11)	
H39B	0.3924	0.8654	0.3604	0.064*	
C40B	0.5193 (3)	0.89365 (14)	0.36853 (15)	0.0457 (10)	
H40B	0.5551	0.8716	0.3558	0.055*	
C41B	1.1125 (3)	0.96382 (17)	0.43597 (14)	0.0498 (11)	
H41D	1.1274	0.9905	0.4506	0.075*	
H41E	1.1354	0.9638	0.4041	0.075*	
H41F	1.1432	0.9414	0.4533	0.075*	
C42B	1.0814 (3)	0.93282 (17)	0.61229 (15)	0.0508 (11)	
H42C	1.1374	0.9417	0.5945	0.061*	
H42D	1.0642	0.9044	0.6022	0.061*	
C43B	1.1059 (4)	0.93229 (19)	0.66253 (17)	0.0587 (13)	
C44B	1.0826 (7)	0.9580 (3)	0.73696 (19)	0.122 (3)	
H44D	1.0856	0.9289	0.7466	0.182*	
H44E	1.0320	0.9721	0.7537	0.182*	
H44F	1.1432	0.9716	0.7438	0.182*	
C45B	0.7084 (2)	0.92626 (11)	0.59904 (12)	0.0301 (7)	
H45C	0.6837	0.9177	0.6295	0.036*	
H45D	0.6996	0.9026	0.5776	0.036*	
C46B	0.6486 (3)	0.96285 (12)	0.58115 (13)	0.0342 (8)	
H46C	0.6496	0.9854	0.6044	0.041*	
H46D	0.6767	0.9737	0.5524	0.041*	
C47B	0.5467 (3)	0.95015 (13)	0.57156 (13)	0.0357 (8)	
C48B	0.4740 (3)	0.96187 (17)	0.60043 (15)	0.0520 (11)	
H48B	0.4877	0.9784	0.6269	0.062*	
C49B	0.3815 (3)	0.9498 (2)	0.59131 (18)	0.0629 (14)	
H49B	0.3328	0.9580	0.6118	0.076*	
C50B	0.3594 (4)	0.92652 (18)	0.55356 (19)	0.0589 (13)	
H50B	0.2957	0.9187	0.5475	0.071*	
C51B	0.4312 (4)	0.91425 (17)	0.52382 (18)	0.0581 (13)	
H51B	0.4172	0.8976	0.4975	0.070*	
C52B	0.5236 (3)	0.92668 (14)	0.53327 (15)	0.0475 (10)	
H52B	0.5722	0.9188	0.5127	0.057*	
C53B	0.8138 (6)	0.9361 (2)	0.74848 (17)	0.107 (3)	
H53D	0.7837	0.9098	0.7570	0.161*	
H53E	0.7722	0.9592	0.7567	0.161*	
H53F	0.8742	0.9389	0.7651	0.161*	
C54B	1.0612 (3)	0.79463 (14)	0.71023 (13)	0.0409 (9)	
H54C	1.0872	0.8039	0.6803	0.049*	
H54D	1.0783	0.7651	0.7145	0.049*	
C55B	1.1053 (4)	0.82009 (16)	0.74905 (17)	0.0551 (12)	
C56B	1.2510 (6)	0.8419 (3)	0.7793 (4)	0.137 (4)	
H56D	1.2421	0.8715	0.7731	0.206*	
H56E	1.3184	0.8350	0.7780	0.206*	
H56F	1.2269	0.8353	0.8101	0.206*	
C57B	0.8434 (2)	0.76435 (11)	0.60507 (11)	0.0258 (7)	
H57C	0.8126	0.7651	0.6356	0.031*	
H57D	0.8022	0.7798	0.5832	0.031*	
C58B	0.8481 (2)	0.71897 (11)	0.58905 (12)	0.0286 (7)	
H58C	0.8839	0.7174	0.5599	0.034*	
H58D	0.8823	0.7023	0.6126	0.034*	
C59B	0.7506 (3)	0.70113 (11)	0.58152 (12)	0.0289 (8)	
C60B	0.6953 (3)	0.68891 (13)	0.61846 (13)	0.0383 (9)	
H60B	0.7206	0.6904	0.6490	0.046*	
C61B	0.6039 (3)	0.67455 (14)	0.61158 (15)	0.0445 (10)	
H61B	0.5671	0.6663	0.6373	0.053*	
C62B	0.5663 (3)	0.67216 (14)	0.56751 (16)	0.0456 (10)	
H62B	0.5035	0.6624	0.5629	0.055*	
C63B	0.6191 (3)	0.68378 (15)	0.53057 (15)	0.0460 (10)	
H63B	0.5938	0.6817	0.5001	0.055*	
C64B	0.7104 (3)	0.69868 (14)	0.53772 (13)	0.0387 (9)	
H64B	0.7462	0.7074	0.5119	0.046*	
C65B	1.2191 (3)	0.82781 (16)	0.60531 (14)	0.0455 (10)	
H65D	1.2583	0.8081	0.5883	0.068*	
H65E	1.2418	0.8296	0.6374	0.068*	
H65F	1.2233	0.8552	0.5908	0.068*	
C66B	1.1609 (3)	0.84164 (12)	0.42021 (13)	0.0347 (8)	
H66C	1.2079	0.8548	0.4411	0.042*	
H66D	1.1048	0.8600	0.4181	0.042*	
C67B	1.2034 (3)	0.83606 (13)	0.37231 (14)	0.0376 (9)	
C68B	1.2921 (4)	0.79220 (17)	0.32324 (16)	0.0632 (14)	
H68D	1.2434	0.7837	0.3008	0.095*	
H68E	1.3390	0.7700	0.3267	0.095*	
H68F	1.3235	0.8174	0.3122	0.095*	
C69B	0.8477 (3)	0.76057 (11)	0.43491 (11)	0.0290 (7)	
H69C	0.7991	0.7784	0.4492	0.035*	
H69D	0.8571	0.7360	0.4550	0.035*	
C70B	0.8125 (3)	0.74636 (15)	0.38724 (13)	0.0461 (11)	
H70C	0.7954	0.7711	0.3686	0.055*	
H70D	0.8644	0.7318	0.3713	0.055*	
C71B	0.7280 (3)	0.71796 (15)	0.38978 (12)	0.0443 (10)	
C72B	0.7407 (4)	0.67666 (16)	0.40247 (16)	0.0561 (13)	
H72B	0.8022	0.6669	0.4102	0.067*	
C73B	0.6645 (4)	0.6497 (2)	0.40400 (19)	0.0738 (17)	
H73B	0.6739	0.6216	0.4129	0.089*	
C74B	0.5767 (4)	0.6633 (2)	0.39283 (18)	0.0747 (18)	
H74B	0.5249	0.6446	0.3937	0.090*	
C75B	0.5619 (4)	0.7033 (2)	0.38035 (16)	0.0666 (16)	
H75B	0.5000	0.7125	0.3723	0.080*	
C76B	0.6388 (4)	0.73129 (18)	0.37936 (15)	0.0568 (13)	
H76B	0.6283	0.7595	0.3714	0.068*	
O1C	0.6334 (2)	0.42533 (8)	0.78498 (8)	0.0386 (6)	
O2C	0.54013 (19)	0.28157 (8)	0.78784 (8)	0.0340 (6)	
O3C	0.4306 (3)	0.31475 (14)	0.71475 (13)	0.0790 (13)	
O4C	0.3017 (3)	0.30626 (12)	0.75901 (13)	0.0643 (10)	
O5C	0.37492 (17)	0.30624 (9)	0.88976 (8)	0.0348 (6)	
O6C	0.36534 (17)	0.30811 (8)	1.05676 (8)	0.0305 (5)	
O7C	0.2863 (3)	0.38220 (12)	1.13619 (12)	0.0658 (10)	
O8C	0.2486 (3)	0.31538 (11)	1.12528 (11)	0.0648 (10)	
O9C	0.5012 (2)	0.29777 (9)	1.15672 (8)	0.0399 (7)	
C54C	0.4775 (4)	0.45404 (17)	1.18003 (18)	0.0640 (14)	
H54E	0.4546	0.4828	1.1750	0.077*	
H54F	0.4239	0.4353	1.1727	0.077*	
O11C	0.4449 (5)	0.4576 (3)	1.2572 (2)	0.167 (3)	
O12C	0.5856 (4)	0.43490 (14)	1.23654 (14)	0.0871 (13)	
O13C	0.43808 (19)	0.45772 (9)	1.04240 (9)	0.0391 (6)	
O14C	0.46610 (18)	0.45251 (9)	0.87540 (9)	0.0355 (6)	
O15C	0.2899 (3)	0.40794 (18)	0.80443 (14)	0.1036 (18)	
O16C	0.3992 (2)	0.45611 (11)	0.79010 (10)	0.0521 (8)	
C1C	0.6202 (2)	0.39033 (11)	0.85618 (11)	0.0265 (7)	
C2C	0.6113 (3)	0.38904 (11)	0.80786 (11)	0.0287 (7)	
C3C	0.5836 (3)	0.35322 (12)	0.78595 (12)	0.0316 (8)	
H3C	0.5769	0.3527	0.7533	0.038*	
C4C	0.5652 (2)	0.31767 (11)	0.81153 (11)	0.0278 (7)	
C5C	0.5737 (2)	0.31754 (10)	0.85965 (11)	0.0241 (7)	
C6C	0.6012 (2)	0.35415 (11)	0.88070 (11)	0.0256 (7)	
H6C	0.6073	0.3547	0.9134	0.031*	
C7C	0.5593 (2)	0.27790 (11)	0.88806 (10)	0.0248 (7)	
H7C	0.5171	0.2591	0.8697	0.030*	
C8C	0.5098 (2)	0.28729 (10)	0.93314 (11)	0.0249 (7)	
C9C	0.4158 (2)	0.30155 (11)	0.93275 (11)	0.0266 (7)	
C10C	0.3688 (2)	0.30952 (11)	0.97340 (12)	0.0282 (7)	
H10C	0.3052	0.3191	0.9724	0.034*	
C11C	0.4141 (2)	0.30364 (11)	1.01567 (11)	0.0263 (7)	
C12C	0.5077 (2)	0.29059 (10)	1.01802 (11)	0.0236 (6)	
C13C	0.5531 (2)	0.28308 (10)	0.97614 (11)	0.0237 (6)	
H13C	0.6174	0.2746	0.9772	0.028*	
C14C	0.5554 (2)	0.28570 (10)	1.06485 (11)	0.0254 (7)	
H14C	0.5138	0.2675	1.0839	0.031*	
C15C	0.5604 (2)	0.32745 (11)	1.08878 (11)	0.0269 (7)	
C16C	0.5293 (3)	0.33302 (11)	1.13396 (11)	0.0293 (7)	
C17C	0.5266 (3)	0.37189 (12)	1.15397 (12)	0.0351 (8)	
H17C	0.5057	0.3751	1.1849	0.042*	
C18C	0.5547 (3)	0.40638 (12)	1.12853 (12)	0.0332 (8)	
C19C	0.5906 (3)	0.40237 (11)	1.08402 (11)	0.0286 (7)	
C20C	0.5910 (2)	0.36275 (11)	1.06521 (11)	0.0266 (7)	
H20C	0.6134	0.3594	1.0346	0.032*	
C21C	0.6269 (3)	0.43926 (11)	1.05680 (11)	0.0290 (7)	
H21C	0.6070	0.4649	1.0736	0.035*	
C22C	0.5836 (2)	0.44144 (10)	1.00860 (11)	0.0276 (7)	
C23C	0.4878 (3)	0.45137 (11)	1.00251 (12)	0.0301 (7)	
C24C	0.4474 (3)	0.45469 (12)	0.95842 (12)	0.0321 (8)	
H24C	0.3824	0.4616	0.9548	0.039*	
C25C	0.5032 (3)	0.44785 (11)	0.92012 (11)	0.0296 (7)	
C26C	0.5989 (2)	0.43753 (10)	0.92381 (11)	0.0252 (7)	
C27C	0.6360 (3)	0.43464 (10)	0.96877 (11)	0.0272 (7)	
H27C	0.7009	0.4276	0.9723	0.033*	
C28C	0.6560 (2)	0.42890 (11)	0.88075 (11)	0.0273 (7)	
H28C	0.6475	0.4530	0.8593	0.033*	
C29C	0.6183 (4)	0.42511 (17)	0.73575 (14)	0.0584 (13)	
H29G	0.6635	0.4061	0.7213	0.088*	
H29H	0.6274	0.4532	0.7236	0.088*	
H29I	0.5536	0.4158	0.7288	0.088*	
C30C	0.4399 (3)	0.27555 (13)	0.78595 (13)	0.0378 (9)	
H30E	0.4123	0.2831	0.8161	0.045*	
H30F	0.4261	0.2459	0.7802	0.045*	
C31C	0.3940 (4)	0.30163 (14)	0.74807 (16)	0.0489 (11)	
C32C	0.2466 (5)	0.3300 (2)	0.7255 (3)	0.103 (3)	
H32G	0.2721	0.3582	0.7234	0.154*	
H32H	0.1805	0.3312	0.7353	0.154*	
H32I	0.2500	0.3165	0.6952	0.154*	
C33C	0.6552 (2)	0.25617 (11)	0.89332 (12)	0.0286 (7)	
H33E	0.6954	0.2728	0.9146	0.034*	
H33F	0.6864	0.2559	0.8628	0.034*	
C34C	0.6513 (3)	0.21142 (11)	0.91156 (12)	0.0312 (8)	
H34E	0.6181	0.2111	0.9415	0.037*	
H34F	0.6145	0.1941	0.8896	0.037*	
C35C	0.7494 (3)	0.19285 (12)	0.91794 (13)	0.0314 (8)	
C36C	0.7943 (4)	0.17242 (16)	0.88213 (15)	0.0532 (12)	
H36C	0.7625	0.1689	0.8533	0.064*	
C37C	0.8855 (4)	0.15704 (17)	0.88807 (17)	0.0632 (14)	
H37C	0.9155	0.1433	0.8632	0.076*	
C38C	0.9323 (3)	0.16145 (15)	0.92905 (18)	0.0532 (12)	
H38C	0.9946	0.1509	0.9328	0.064*	
C39C	0.8886 (3)	0.18131 (15)	0.96496 (17)	0.0512 (11)	
H39C	0.9203	0.1842	0.9939	0.061*	
C40C	0.7975 (3)	0.19727 (14)	0.95899 (14)	0.0434 (10)	
H40C	0.7684	0.2115	0.9838	0.052*	
C41C	0.2804 (3)	0.32222 (15)	0.88786 (15)	0.0456 (10)	
H41G	0.2375	0.3027	0.9030	0.068*	
H41H	0.2608	0.3258	0.8555	0.068*	
H41I	0.2781	0.3491	0.9037	0.068*	
C42C	0.3361 (3)	0.34942 (12)	1.06685 (13)	0.0355 (8)	
H42E	0.2914	0.3591	1.0426	0.043*	
H42F	0.3918	0.3681	1.0669	0.043*	
C43C	0.2889 (3)	0.35114 (14)	1.11333 (14)	0.0444 (10)	
C44C	0.1966 (5)	0.3146 (2)	1.1685 (2)	0.089 (2)	
H44G	0.2370	0.3252	1.1935	0.134*	
H44H	0.1775	0.2861	1.1754	0.134*	
H44I	0.1401	0.3321	1.1655	0.134*	
C45C	0.6529 (3)	0.26473 (12)	1.06296 (12)	0.0321 (8)	
H45E	0.6479	0.2392	1.0440	0.039*	
H45F	0.6980	0.2837	1.0477	0.039*	
C46C	0.6913 (3)	0.25344 (15)	1.11070 (13)	0.0456 (10)	
H46E	0.7061	0.2792	1.1279	0.055*	
H46F	0.6419	0.2383	1.1279	0.055*	
C47C	0.7798 (3)	0.22670 (15)	1.10839 (12)	0.0452 (11)	
C48C	0.7739 (4)	0.18567 (16)	1.09488 (16)	0.0540 (12)	
H48C	0.7140	0.1742	1.0869	0.065*	
C49C	0.8535 (4)	0.16114 (19)	1.09284 (19)	0.0649 (14)	
H49C	0.8488	0.1333	1.0824	0.078*	
C50C	0.9399 (4)	0.1771 (2)	1.10603 (18)	0.0634 (14)	
H50C	0.9945	0.1600	1.1058	0.076*	
C51C	0.9474 (3)	0.21738 (18)	1.11938 (14)	0.0526 (12)	
H51C	1.0071	0.2285	1.1282	0.063*	
C52C	0.8672 (3)	0.24233 (17)	1.12010 (14)	0.0515 (11)	
H52C	0.8730	0.2706	1.1288	0.062*	
C53C	0.4736 (5)	0.30092 (18)	1.20303 (13)	0.0723 (19)	
H53G	0.5256	0.3127	1.2214	0.108*	
H53H	0.4578	0.2733	1.2148	0.108*	
H53I	0.4180	0.3190	1.2051	0.108*	
O10C	0.5506 (2)	0.44617 (9)	1.14663 (9)	0.0484 (7)	
C55C	0.4986 (5)	0.4497 (2)	1.2281 (2)	0.0775 (18)	
C56C	0.6140 (7)	0.4280 (3)	1.2873 (2)	0.127 (3)	
H56G	0.6258	0.4549	1.3021	0.190*	
H56H	0.6715	0.4111	1.2889	0.190*	
H56I	0.5627	0.4137	1.3034	0.190*	
C57C	0.7351 (3)	0.43904 (12)	1.05619 (12)	0.0320 (8)	
H57E	0.7568	0.4133	1.0410	0.038*	
H57F	0.7572	0.4628	1.0375	0.038*	
C58C	0.7801 (3)	0.44175 (13)	1.10428 (13)	0.0412 (9)	
H58E	0.7643	0.4690	1.1180	0.049*	
H58F	0.7521	0.4200	1.1241	0.049*	
C59C	0.8861 (3)	0.43667 (13)	1.10470 (15)	0.0457 (11)	
C60C	0.9422 (3)	0.45302 (15)	1.07105 (19)	0.0584 (13)	
H60C	0.9136	0.4687	1.0468	0.070*	
C61C	1.0395 (4)	0.44750 (17)	1.0711 (2)	0.0699 (16)	
H61C	1.0767	0.4594	1.0473	0.084*	
C62C	1.0818 (4)	0.42485 (19)	1.1057 (3)	0.081 (2)	
H62C	1.1482	0.4202	1.1057	0.097*	
C63C	1.0268 (5)	0.4090 (2)	1.1403 (2)	0.081 (2)	
H63C	1.0559	0.3944	1.1651	0.097*	
C64C	0.9305 (4)	0.41407 (17)	1.13970 (17)	0.0653 (15)	
H64C	0.8936	0.4020	1.1634	0.078*	
C65C	0.3383 (3)	0.46367 (17)	1.03865 (16)	0.0518 (11)	
H65G	0.3252	0.4907	1.0245	0.078*	
H65H	0.3103	0.4627	1.0695	0.078*	
H65I	0.3107	0.4417	1.0194	0.078*	
C66C	0.3882 (4)	0.4269 (2)	0.86587 (16)	0.080 (2)	
H66E	0.3356	0.4348	0.8864	0.096*	
H66F	0.4056	0.3978	0.8731	0.096*	
C67C	0.3544 (4)	0.42902 (19)	0.81719 (16)	0.0621 (14)	
C68C	0.3666 (4)	0.4575 (2)	0.74228 (15)	0.0662 (15)	
H68G	0.3594	0.4291	0.7305	0.099*	
H68H	0.4129	0.4724	0.7236	0.099*	
H68I	0.3055	0.4718	0.7406	0.099*	
C69C	0.7634 (2)	0.42304 (11)	0.88933 (12)	0.0311 (8)	
H69E	0.7720	0.4018	0.9137	0.037*	
H69F	0.7915	0.4118	0.8607	0.037*	
C70C	0.8198 (3)	0.46189 (12)	0.90363 (14)	0.0356 (8)	
H70E	0.8090	0.4844	0.8809	0.043*	
H70F	0.7987	0.4717	0.9342	0.043*	
C71C	0.9229 (3)	0.45106 (13)	0.90571 (16)	0.0449 (10)	
C72C	0.9594 (3)	0.42801 (17)	0.9421 (2)	0.0673 (15)	
H72C	0.9192	0.4208	0.9669	0.081*	
C73C	1.0525 (5)	0.4152 (2)	0.9431 (3)	0.110 (3)	
H73C	1.0771	0.4000	0.9687	0.132*	
C74C	1.1092 (4)	0.4251 (2)	0.9060 (5)	0.132 (4)	
H74C	1.1727	0.4156	0.9056	0.159*	
C75C	1.0752 (5)	0.4489 (3)	0.8691 (3)	0.109 (3)	
H75C	1.1156	0.4562	0.8444	0.131*	
C76C	0.9810 (4)	0.46159 (19)	0.8690 (2)	0.0682 (15)	
H76C	0.9565	0.4774	0.8440	0.082*	
O1D	0.8576 (2)	0.57303 (8)	0.85863 (8)	0.0347 (6)	
O2D	0.76530 (17)	0.71659 (8)	0.86430 (8)	0.0314 (5)	
O3D	0.6548 (3)	0.68559 (14)	0.78997 (11)	0.0698 (11)	
O4D	0.5299 (2)	0.68868 (11)	0.83635 (12)	0.0603 (9)	
O5D	0.60316 (16)	0.69184 (8)	0.96648 (8)	0.0309 (5)	
O6D	0.59365 (17)	0.69021 (7)	1.13331 (8)	0.0283 (5)	
O7D	0.4997 (2)	0.61489 (10)	1.20839 (11)	0.0564 (8)	
O8D	0.4720 (2)	0.68293 (9)	1.20022 (10)	0.0488 (7)	
O9D	0.7279 (2)	0.69822 (10)	1.23279 (9)	0.0460 (8)	
O10D	0.7668 (2)	0.54882 (10)	1.21833 (9)	0.0466 (7)	
O11D	0.6741 (4)	0.5312 (2)	1.33062 (14)	0.130 (2)	
O12D	0.8111 (3)	0.55522 (15)	1.30678 (11)	0.0768 (12)	
O13D	0.65440 (18)	0.54636 (9)	1.11335 (8)	0.0343 (6)	
O14D	0.69516 (16)	0.54863 (8)	0.94702 (7)	0.0293 (5)	
O15D	0.4968 (3)	0.56939 (16)	0.87660 (11)	0.0835 (14)	
O16D	0.62933 (19)	0.53456 (9)	0.86243 (8)	0.0390 (6)	
C1D	0.8491 (2)	0.60721 (11)	0.93081 (10)	0.0242 (7)	
C2D	0.8376 (2)	0.60882 (11)	0.88217 (11)	0.0256 (7)	
C3D	0.8090 (2)	0.64512 (11)	0.86101 (11)	0.0282 (7)	
H3D	0.8011	0.6461	0.8284	0.034*	
C4D	0.7917 (2)	0.68037 (11)	0.88744 (11)	0.0258 (7)	
C5D	0.8012 (2)	0.68024 (11)	0.93557 (11)	0.0244 (7)	
C6D	0.8304 (2)	0.64293 (10)	0.95560 (10)	0.0238 (7)	
H6D	0.8379	0.6420	0.9882	0.029*	
C7D	0.7879 (2)	0.71927 (10)	0.96432 (10)	0.0235 (6)	
H7D	0.7455	0.7383	0.9463	0.028*	
C8D	0.7386 (2)	0.70960 (10)	1.00988 (10)	0.0226 (6)	
C9D	0.6447 (2)	0.69641 (11)	1.00945 (11)	0.0256 (7)	
C10D	0.5967 (2)	0.68893 (11)	1.05020 (11)	0.0266 (7)	
H10D	0.5326	0.6800	1.0494	0.032*	
C11D	0.6433 (2)	0.69465 (10)	1.09212 (11)	0.0249 (7)	
C12D	0.7368 (2)	0.70689 (10)	1.09453 (11)	0.0246 (7)	
C13D	0.7833 (2)	0.71382 (10)	1.05297 (11)	0.0242 (7)	
H13D	0.8480	0.7217	1.0540	0.029*	
C14D	0.7859 (2)	0.71099 (11)	1.14178 (11)	0.0269 (7)	
H14D	0.7451	0.7293	1.1611	0.032*	
C15D	0.7879 (2)	0.66872 (12)	1.16476 (10)	0.0271 (7)	
C16D	0.7543 (3)	0.66291 (13)	1.20978 (11)	0.0330 (8)	
C17D	0.7475 (3)	0.62339 (13)	1.22849 (12)	0.0377 (9)	
H17D	0.7246	0.6197	1.2590	0.045*	
C18D	0.7746 (3)	0.58907 (13)	1.20216 (12)	0.0353 (8)	
C19D	0.8114 (2)	0.59367 (11)	1.15822 (11)	0.0275 (7)	
C20D	0.8168 (2)	0.63369 (11)	1.14096 (10)	0.0261 (7)	
H20D	0.8418	0.6374	1.1109	0.031*	
C21D	0.8465 (3)	0.55645 (11)	1.13036 (11)	0.0291 (7)	
H21D	0.8230	0.5309	1.1461	0.035*	
C22D	0.8051 (2)	0.55628 (10)	1.08138 (10)	0.0262 (7)	
C23D	0.7085 (2)	0.55013 (11)	1.07421 (11)	0.0273 (7)	
C24D	0.6699 (2)	0.54738 (11)	1.02982 (11)	0.0268 (7)	
H24D	0.6041	0.5427	1.0256	0.032*	
C25D	0.7285 (2)	0.55150 (11)	0.99182 (11)	0.0240 (7)	
C26D	0.8247 (2)	0.55976 (11)	0.99720 (11)	0.0241 (7)	
C27D	0.8603 (2)	0.56165 (10)	1.04224 (11)	0.0252 (7)	
H27D	0.9259	0.5669	1.0465	0.030*	
C28D	0.8848 (2)	0.56808 (11)	0.95450 (11)	0.0246 (7)	
H28D	0.8763	0.5443	0.9326	0.030*	
C29D	0.8370 (3)	0.57293 (14)	0.81022 (12)	0.0431 (10)	
H29J	0.7715	0.5819	0.8050	0.065*	
H29K	0.8453	0.5448	0.7980	0.065*	
H29L	0.8799	0.5920	0.7945	0.065*	
C30D	0.6645 (3)	0.72198 (13)	0.86260 (13)	0.0355 (8)	
H30G	0.6498	0.7516	0.8572	0.043*	
H30H	0.6375	0.7140	0.8927	0.043*	
C31D	0.6195 (3)	0.69637 (13)	0.82514 (15)	0.0428 (10)	
C32D	0.4737 (5)	0.6647 (2)	0.8036 (2)	0.090 (2)	
H32J	0.4677	0.6802	0.7745	0.136*	
H32K	0.4107	0.6598	0.8163	0.136*	
H32L	0.5049	0.6381	0.7978	0.136*	
C33D	0.8841 (2)	0.74125 (11)	0.97000 (11)	0.0270 (7)	
H33G	0.9240	0.7247	0.9914	0.032*	
H33H	0.9158	0.7415	0.9397	0.032*	
C34D	0.8800 (2)	0.78596 (12)	0.98813 (12)	0.0304 (7)	
H34G	0.8448	0.8035	0.9658	0.036*	
H34H	0.8457	0.7865	1.0178	0.036*	
C35D	0.9782 (3)	0.80352 (11)	0.99530 (13)	0.0301 (8)	
C36D	1.0259 (3)	0.82269 (13)	0.95902 (14)	0.0419 (9)	
H36D	0.9954	0.8263	0.9299	0.050*	
C37D	1.1190 (3)	0.83655 (15)	0.96579 (16)	0.0513 (11)	
H37D	1.1519	0.8489	0.9408	0.062*	
C38D	1.1633 (3)	0.83252 (14)	1.00781 (17)	0.0482 (11)	
H38D	1.2261	0.8423	1.0120	0.058*	
C39D	1.1164 (3)	0.81424 (15)	1.04392 (16)	0.0477 (10)	
H39D	1.1469	0.8115	1.0732	0.057*	
C40D	1.0241 (3)	0.79977 (14)	1.03774 (14)	0.0397 (9)	
H40D	0.9923	0.7872	1.0629	0.048*	
C41D	0.5119 (3)	0.67344 (15)	0.96403 (13)	0.0423 (10)	
H41J	0.5134	0.6464	0.9795	0.063*	
H41K	0.4933	0.6697	0.9316	0.063*	
H41L	0.4661	0.6915	0.9793	0.063*	
C42D	0.5611 (3)	0.64925 (11)	1.14234 (13)	0.0320 (8)	
H42G	0.6157	0.6300	1.1432	0.038*	
H42H	0.5182	0.6403	1.1170	0.038*	
C43D	0.5093 (3)	0.64697 (13)	1.18770 (13)	0.0371 (9)	
C44D	0.4151 (4)	0.68332 (18)	1.24156 (17)	0.0702 (16)	
H44J	0.3552	0.6689	1.2355	0.105*	
H44K	0.4022	0.7121	1.2505	0.105*	
H44L	0.4493	0.6693	1.2666	0.105*	
C45D	0.8834 (3)	0.73162 (12)	1.13942 (11)	0.0304 (8)	
H45G	0.9279	0.7127	1.1237	0.037*	
H45H	0.8785	0.7573	1.1208	0.037*	
C46D	0.9228 (3)	0.74229 (16)	1.18744 (13)	0.0471 (11)	
H46G	0.8728	0.7561	1.2056	0.057*	
H46H	0.9406	0.7164	1.2037	0.057*	
C47D	1.0080 (3)	0.77037 (16)	1.18500 (12)	0.0456 (11)	
C48D	0.9991 (4)	0.81137 (17)	1.17239 (15)	0.0546 (12)	
H48D	0.9384	0.8216	1.1637	0.065*	
C49D	1.0755 (4)	0.83820 (19)	1.17188 (18)	0.0640 (14)	
H49D	1.0672	0.8665	1.1634	0.077*	
C50D	1.1646 (4)	0.8232 (2)	1.18391 (16)	0.0617 (14)	
H50D	1.2179	0.8411	1.1833	0.074*	
C51D	1.1753 (3)	0.78279 (19)	1.19651 (14)	0.0546 (13)	
H51D	1.2360	0.7727	1.2054	0.066*	
C52D	1.0980 (3)	0.75608 (17)	1.19643 (13)	0.0500 (11)	
H52D	1.1068	0.7277	1.2043	0.060*	
C53D	0.6941 (5)	0.69408 (19)	1.27797 (13)	0.0715 (18)	
H53J	0.6363	0.6773	1.2776	0.107*	
H53K	0.6802	0.7216	1.2906	0.107*	
H53L	0.7422	0.6804	1.2973	0.107*	
C54D	0.6951 (4)	0.54023 (18)	1.25043 (16)	0.0583 (13)	
H54G	0.6703	0.5121	1.2440	0.070*	
H54H	0.6427	0.5601	1.2451	0.070*	
C55D	0.7230 (4)	0.54227 (17)	1.30033 (16)	0.0599 (13)	
C56D	0.8448 (6)	0.5573 (3)	1.3548 (2)	0.110 (3)	
H56J	0.8061	0.5771	1.3720	0.165*	
H56K	0.9110	0.5664	1.3554	0.165*	
H56L	0.8400	0.5298	1.3690	0.165*	
C57D	0.9557 (3)	0.55471 (12)	1.13162 (11)	0.0329 (8)	
H57G	0.9777	0.5333	1.1096	0.039*	
H57H	0.9814	0.5818	1.1216	0.039*	
C58D	0.9938 (3)	0.54457 (18)	1.17988 (14)	0.0523 (12)	
H58G	0.9627	0.5629	1.2026	0.063*	
H58H	0.9767	0.5156	1.1876	0.063*	
C59D	1.0996 (3)	0.54957 (14)	1.18446 (13)	0.0436 (10)	
C60D	1.1596 (4)	0.52542 (19)	1.1591 (2)	0.0725 (17)	
H60D	1.1342	0.5057	1.1381	0.087*	
C61D	1.2574 (5)	0.5293 (3)	1.1637 (2)	0.099 (2)	
H61D	1.2975	0.5113	1.1469	0.119*	
C62D	1.2964 (4)	0.5588 (2)	1.1921 (2)	0.0765 (18)	
H62D	1.3630	0.5625	1.1941	0.092*	
C63D	1.2372 (5)	0.5823 (2)	1.2172 (2)	0.0758 (18)	
H63D	1.2630	0.6023	1.2379	0.091*	
C64D	1.1388 (4)	0.57823 (18)	1.21380 (16)	0.0617 (14)	
H64D	1.0991	0.5954	1.2319	0.074*	
C65D	0.5544 (3)	0.54612 (13)	1.10817 (13)	0.0381 (8)	
H65J	0.5347	0.5696	1.0888	0.057*	
H65K	0.5251	0.5485	1.1386	0.057*	
H65L	0.5344	0.5201	1.0935	0.057*	
C66D	0.5971 (3)	0.55178 (15)	0.94016 (13)	0.0438 (10)	
H66G	0.5737	0.5771	0.9558	0.053*	
H66H	0.5658	0.5275	0.9544	0.053*	
C67D	0.5703 (3)	0.55367 (16)	0.88947 (13)	0.0485 (11)	
C68D	0.5984 (4)	0.53020 (16)	0.81441 (14)	0.0506 (12)	
H68J	0.5426	0.5122	0.8129	0.076*	
H68K	0.6495	0.5179	0.7963	0.076*	
H68L	0.5825	0.5576	0.8018	0.076*	
C69D	0.9912 (2)	0.57360 (11)	0.96396 (12)	0.0302 (7)	
H69G	1.0200	0.5850	0.9356	0.036*	
H69H	0.9987	0.5948	0.9885	0.036*	
C70D	1.0484 (3)	0.53524 (13)	0.97847 (14)	0.0396 (9)	
H70G	1.0230	0.5239	1.0076	0.047*	
H70H	1.0425	0.5135	0.9544	0.047*	
C71D	1.1510 (3)	0.54668 (14)	0.98517 (17)	0.0455 (10)	
C72D	1.2143 (4)	0.5444 (2)	0.9493 (2)	0.0774 (19)	
H72D	1.1956	0.5325	0.9206	0.093*	
C73D	1.3082 (5)	0.5601 (3)	0.9557 (4)	0.114 (3)	
H73D	1.3522	0.5596	0.9311	0.137*	
C74D	1.3337 (6)	0.5760 (3)	0.9986 (5)	0.129 (4)	
H74D	1.3964	0.5860	1.0030	0.155*	
C75D	1.2723 (5)	0.5779 (2)	1.0348 (4)	0.104 (3)	
H75D	1.2912	0.5887	1.0640	0.125*	
C76D	1.1820 (4)	0.56335 (17)	1.0270 (2)	0.0694 (15)	
H76D	1.1382	0.5648	1.0517	0.083*	

### Atomic displacement parameters (Å^2^)

**Table:**

	*U*^11^	*U*^22^	*U*^33^	*U*^12^	*U*^13^	*U*^23^
O1A	0.0616 (19)	0.0339 (15)	0.0263 (12)	−0.0052 (13)	0.0100 (12)	0.0060 (11)
O2A	0.0409 (15)	0.0331 (14)	0.0295 (12)	−0.0001 (11)	−0.0042 (11)	−0.0062 (11)
O3A	0.065 (2)	0.097 (3)	0.060 (2)	0.000 (2)	−0.0179 (18)	0.033 (2)
O4A	0.046 (2)	0.067 (2)	0.089 (3)	−0.0014 (17)	−0.0245 (18)	0.008 (2)
O5A	0.0232 (13)	0.0501 (17)	0.0326 (12)	0.0078 (12)	−0.0048 (10)	0.0064 (11)
O6A	0.0315 (13)	0.0301 (13)	0.0320 (12)	0.0043 (10)	0.0110 (10)	0.0004 (10)
O7A	0.070 (2)	0.053 (2)	0.0483 (17)	−0.0019 (17)	0.0149 (15)	−0.0148 (15)
O8A	0.0508 (17)	0.0421 (17)	0.0420 (15)	−0.0003 (14)	0.0229 (13)	−0.0021 (12)
O9A	0.0522 (17)	0.0398 (15)	0.0232 (12)	−0.0069 (13)	0.0107 (11)	0.0041 (11)
O10A	0.0555 (18)	0.0407 (16)	0.0272 (12)	−0.0047 (13)	0.0108 (11)	−0.0080 (11)
O11A	0.066 (3)	0.110 (4)	0.105 (3)	0.031 (2)	0.014 (2)	0.053 (3)
O12A	0.052 (2)	0.087 (3)	0.0577 (19)	0.0113 (18)	0.0157 (15)	0.0225 (18)
O13A	0.0365 (15)	0.0507 (17)	0.0360 (13)	0.0109 (13)	0.0164 (11)	−0.0010 (12)
O14A	0.0254 (12)	0.0317 (13)	0.0342 (12)	−0.0006 (10)	0.0006 (10)	0.0061 (10)
O15A	0.093 (3)	0.117 (4)	0.068 (2)	−0.058 (3)	−0.039 (2)	0.026 (2)
O16A	0.0553 (18)	0.0403 (17)	0.0386 (14)	−0.0041 (14)	−0.0009 (12)	0.0023 (12)
C1A	0.0263 (17)	0.0243 (17)	0.0242 (15)	0.0037 (13)	0.0028 (12)	0.0003 (12)
C2A	0.036 (2)	0.0258 (18)	0.0258 (16)	0.0054 (15)	0.0037 (14)	0.0038 (13)
C3A	0.038 (2)	0.036 (2)	0.0220 (15)	0.0094 (16)	0.0015 (13)	0.0049 (14)
C4A	0.0330 (19)	0.0270 (18)	0.0241 (15)	0.0043 (14)	−0.0024 (13)	−0.0020 (13)
C5A	0.0229 (16)	0.0257 (17)	0.0246 (15)	0.0066 (13)	0.0028 (12)	0.0004 (12)
C6A	0.0228 (16)	0.0276 (17)	0.0219 (14)	0.0047 (13)	0.0006 (12)	0.0036 (12)
C7A	0.0224 (16)	0.0267 (18)	0.0215 (14)	−0.0003 (13)	0.0002 (12)	0.0021 (12)
C8A	0.0234 (17)	0.0210 (16)	0.0276 (15)	−0.0009 (13)	−0.0001 (12)	0.0021 (12)
C9A	0.0228 (16)	0.0268 (17)	0.0254 (15)	−0.0013 (13)	−0.0015 (12)	0.0015 (13)
C10A	0.0200 (16)	0.0336 (19)	0.0331 (17)	−0.0038 (14)	0.0013 (13)	−0.0002 (14)
C11A	0.0224 (16)	0.0240 (17)	0.0275 (15)	−0.0058 (13)	0.0061 (12)	−0.0001 (13)
C12A	0.0276 (17)	0.0210 (16)	0.0258 (15)	−0.0007 (13)	0.0027 (13)	0.0000 (12)
C13A	0.0227 (16)	0.0249 (17)	0.0254 (15)	−0.0016 (13)	0.0039 (12)	0.0010 (12)
C14A	0.0306 (18)	0.0267 (18)	0.0211 (14)	0.0041 (14)	0.0043 (12)	0.0020 (12)
C15A	0.0249 (17)	0.0290 (18)	0.0214 (14)	0.0026 (13)	0.0011 (12)	0.0009 (13)
C16A	0.0261 (17)	0.036 (2)	0.0228 (15)	0.0038 (14)	0.0034 (13)	0.0057 (13)
C17A	0.0334 (19)	0.040 (2)	0.0195 (14)	0.0060 (16)	0.0042 (13)	0.0005 (14)
C18A	0.0341 (19)	0.034 (2)	0.0252 (16)	0.0039 (15)	0.0037 (14)	−0.0045 (14)
C19A	0.0284 (18)	0.0314 (19)	0.0238 (15)	0.0036 (14)	0.0060 (13)	0.0022 (13)
C20A	0.0292 (18)	0.0310 (18)	0.0203 (14)	0.0050 (14)	0.0052 (12)	0.0011 (13)
C21A	0.035 (2)	0.0307 (19)	0.0229 (15)	0.0027 (15)	0.0045 (13)	−0.0037 (14)
C22A	0.0338 (19)	0.0231 (17)	0.0244 (15)	0.0008 (14)	0.0049 (13)	−0.0012 (12)
C23A	0.0334 (19)	0.0254 (17)	0.0329 (17)	0.0020 (15)	0.0147 (14)	0.0012 (14)
C24A	0.0241 (17)	0.0258 (18)	0.0389 (18)	0.0025 (14)	0.0045 (14)	0.0030 (14)
C25A	0.0294 (18)	0.0206 (16)	0.0285 (16)	0.0029 (14)	0.0027 (13)	0.0049 (13)
C26A	0.0235 (16)	0.0210 (17)	0.0282 (15)	0.0003 (13)	0.0051 (13)	0.0011 (13)
C27A	0.0253 (17)	0.0218 (16)	0.0281 (16)	0.0020 (13)	0.0033 (13)	−0.0004 (13)
C28A	0.0287 (17)	0.0241 (17)	0.0227 (14)	0.0011 (14)	0.0067 (12)	0.0007 (12)
C29A	0.119 (5)	0.066 (3)	0.027 (2)	−0.030 (3)	0.011 (2)	0.005 (2)
C30A	0.036 (2)	0.042 (2)	0.0353 (19)	−0.0046 (17)	−0.0044 (16)	−0.0043 (16)
C31A	0.049 (3)	0.046 (3)	0.047 (2)	−0.002 (2)	−0.017 (2)	0.000 (2)
C32A	0.073 (4)	0.094 (5)	0.135 (6)	0.008 (4)	−0.052 (4)	0.028 (5)
C33A	0.0304 (18)	0.0241 (17)	0.0285 (16)	0.0034 (14)	0.0034 (13)	0.0043 (13)
C34A	0.0307 (19)	0.0257 (18)	0.0351 (18)	0.0011 (15)	0.0023 (14)	0.0007 (14)
C35A	0.0308 (19)	0.0228 (17)	0.0327 (17)	−0.0005 (14)	0.0014 (14)	0.0020 (14)
C36A	0.041 (2)	0.035 (2)	0.0365 (19)	0.0097 (17)	0.0041 (16)	−0.0005 (16)
C37A	0.045 (2)	0.039 (2)	0.052 (2)	0.0129 (19)	0.017 (2)	0.0047 (19)
C38A	0.030 (2)	0.039 (2)	0.066 (3)	0.0053 (17)	0.0024 (19)	0.012 (2)
C39A	0.037 (2)	0.052 (3)	0.044 (2)	0.0019 (19)	−0.0063 (18)	0.0036 (19)
C40A	0.036 (2)	0.049 (3)	0.0348 (19)	0.0043 (18)	0.0003 (16)	−0.0030 (18)
C41A	0.026 (2)	0.064 (3)	0.040 (2)	0.0049 (19)	−0.0072 (16)	0.0056 (19)
C42A	0.031 (2)	0.033 (2)	0.0361 (18)	0.0053 (16)	0.0028 (15)	−0.0012 (15)
C43A	0.036 (2)	0.044 (2)	0.0338 (18)	0.0089 (18)	0.0039 (15)	−0.0041 (17)
C44A	0.076 (4)	0.057 (3)	0.049 (3)	0.004 (3)	0.031 (2)	0.006 (2)
C45A	0.0333 (19)	0.0297 (19)	0.0240 (15)	0.0063 (15)	−0.0011 (13)	0.0007 (13)
C46A	0.053 (3)	0.061 (3)	0.0273 (18)	0.026 (2)	0.0011 (17)	0.0036 (18)
C47A	0.049 (2)	0.055 (3)	0.0234 (17)	0.019 (2)	0.0064 (16)	0.0111 (17)
C48A	0.053 (3)	0.067 (3)	0.0286 (19)	−0.001 (2)	0.0096 (18)	0.0141 (19)
C49A	0.040 (3)	0.097 (4)	0.035 (2)	0.006 (3)	0.0115 (18)	0.019 (2)
C50A	0.053 (3)	0.082 (4)	0.060 (3)	0.027 (3)	0.007 (2)	0.004 (3)
C51A	0.068 (4)	0.056 (3)	0.075 (3)	0.023 (3)	0.007 (3)	0.000 (3)
C52A	0.044 (3)	0.060 (3)	0.057 (3)	0.012 (2)	0.002 (2)	0.001 (2)
C53A	0.087 (4)	0.063 (3)	0.0236 (18)	−0.026 (3)	0.011 (2)	0.0047 (19)
C54A	0.046 (2)	0.045 (2)	0.0312 (18)	0.0036 (19)	0.0042 (16)	−0.0079 (16)
C55A	0.057 (3)	0.037 (2)	0.048 (2)	0.013 (2)	0.018 (2)	0.0044 (19)
C56A	0.056 (3)	0.092 (4)	0.079 (4)	0.010 (3)	0.022 (3)	0.011 (3)
C57A	0.0343 (19)	0.038 (2)	0.0249 (15)	−0.0016 (16)	0.0021 (13)	−0.0023 (14)
C58A	0.043 (2)	0.057 (3)	0.0262 (17)	0.003 (2)	−0.0016 (15)	−0.0044 (17)
C59A	0.038 (2)	0.051 (2)	0.0246 (16)	−0.0087 (18)	−0.0034 (14)	−0.0052 (16)
C60A	0.041 (2)	0.061 (3)	0.042 (2)	−0.011 (2)	−0.0067 (17)	0.009 (2)
C61A	0.040 (3)	0.084 (4)	0.059 (3)	0.007 (3)	−0.011 (2)	0.007 (3)
C62A	0.047 (3)	0.101 (5)	0.044 (3)	−0.013 (3)	−0.006 (2)	0.001 (3)
C63A	0.069 (4)	0.092 (5)	0.046 (3)	−0.048 (3)	0.003 (2)	−0.007 (3)
C64A	0.063 (3)	0.048 (3)	0.045 (2)	−0.014 (2)	−0.005 (2)	−0.0042 (19)
C65A	0.040 (2)	0.043 (2)	0.053 (2)	0.0069 (18)	0.0228 (19)	0.0059 (18)
C66A	0.037 (2)	0.073 (3)	0.041 (2)	−0.015 (2)	−0.0050 (17)	0.010 (2)
C67A	0.049 (3)	0.057 (3)	0.045 (2)	−0.012 (2)	−0.0112 (19)	0.009 (2)
C68A	0.100 (4)	0.056 (3)	0.042 (2)	−0.011 (3)	−0.008 (3)	0.000 (2)
C69A	0.0291 (18)	0.0290 (18)	0.0306 (17)	0.0011 (15)	0.0042 (13)	−0.0018 (14)
C70A	0.0302 (19)	0.032 (2)	0.0411 (19)	0.0009 (15)	0.0042 (15)	−0.0011 (15)
C71A	0.033 (2)	0.038 (2)	0.0382 (19)	−0.0004 (17)	0.0006 (15)	−0.0072 (16)
C72A	0.045 (2)	0.051 (3)	0.046 (2)	0.006 (2)	0.0045 (18)	0.0000 (19)
C73A	0.047 (3)	0.064 (3)	0.053 (3)	0.010 (2)	−0.014 (2)	−0.004 (2)
C74A	0.036 (2)	0.074 (4)	0.076 (3)	0.018 (2)	−0.012 (2)	−0.025 (3)
C75A	0.035 (2)	0.099 (4)	0.055 (3)	0.006 (3)	0.003 (2)	−0.019 (3)
C76A	0.036 (2)	0.077 (3)	0.040 (2)	0.000 (2)	0.0025 (17)	−0.002 (2)
O1B	0.0579 (18)	0.0345 (15)	0.0222 (12)	0.0081 (13)	0.0111 (11)	−0.0023 (10)
O2B	0.0559 (18)	0.0388 (15)	0.0286 (12)	0.0081 (13)	0.0062 (11)	0.0121 (11)
O3B	0.061 (2)	0.086 (3)	0.0489 (18)	−0.0269 (19)	0.0013 (15)	−0.0055 (17)
O4B	0.058 (2)	0.124 (4)	0.0452 (18)	−0.002 (2)	0.0161 (15)	−0.020 (2)
O5B	0.0315 (14)	0.0574 (18)	0.0308 (13)	−0.0069 (13)	0.0056 (10)	0.0017 (12)
O6B	0.0348 (14)	0.0482 (17)	0.0304 (12)	−0.0007 (12)	−0.0034 (10)	−0.0035 (11)
O7B	0.105 (4)	0.153 (5)	0.063 (3)	0.058 (4)	−0.028 (2)	−0.006 (3)
O8B	0.126 (4)	0.063 (2)	0.0412 (18)	0.011 (2)	−0.0184 (19)	−0.0105 (17)
O9B	0.085 (2)	0.0377 (16)	0.0248 (12)	0.0183 (15)	0.0048 (13)	−0.0054 (11)
O10B	0.0358 (14)	0.0343 (14)	0.0266 (12)	0.0050 (11)	−0.0064 (10)	0.0053 (10)
O11B	0.082 (3)	0.104 (3)	0.061 (2)	0.013 (2)	−0.029 (2)	−0.035 (2)
O12B	0.056 (2)	0.069 (3)	0.111 (3)	0.0037 (19)	−0.041 (2)	−0.010 (2)
O13B	0.0230 (13)	0.0492 (17)	0.0305 (12)	−0.0025 (11)	−0.0046 (10)	0.0001 (11)
O14B	0.0362 (14)	0.0348 (14)	0.0294 (12)	0.0006 (11)	0.0091 (10)	0.0019 (10)
O15B	0.060 (2)	0.059 (2)	0.0388 (15)	−0.0052 (16)	0.0061 (13)	0.0166 (14)
O16B	0.0568 (19)	0.0422 (17)	0.0410 (15)	−0.0007 (14)	0.0209 (13)	0.0003 (13)
C1B	0.0244 (17)	0.0276 (18)	0.0230 (15)	−0.0043 (13)	0.0007 (12)	−0.0012 (13)
C2B	0.0287 (18)	0.0316 (19)	0.0231 (15)	−0.0024 (14)	0.0009 (13)	−0.0037 (13)
C3B	0.0361 (19)	0.0346 (19)	0.0195 (14)	0.0010 (15)	0.0015 (13)	0.0025 (13)
C4B	0.0297 (18)	0.0307 (19)	0.0262 (16)	0.0014 (14)	0.0002 (13)	0.0056 (13)
C5B	0.0301 (18)	0.0283 (18)	0.0230 (15)	−0.0013 (14)	0.0013 (13)	0.0010 (13)
C6B	0.0239 (17)	0.0293 (18)	0.0223 (14)	0.0001 (13)	0.0018 (12)	0.0010 (12)
C7B	0.0288 (17)	0.0232 (17)	0.0258 (15)	0.0035 (14)	0.0016 (13)	0.0037 (13)
C8B	0.0318 (18)	0.0198 (16)	0.0259 (15)	0.0019 (14)	−0.0009 (13)	−0.0006 (12)
C9B	0.0284 (18)	0.0314 (19)	0.0291 (16)	−0.0007 (14)	0.0041 (13)	0.0031 (14)
C10B	0.0263 (18)	0.036 (2)	0.0384 (18)	−0.0064 (15)	0.0036 (14)	0.0036 (15)
C11B	0.0304 (19)	0.0296 (19)	0.0299 (17)	0.0032 (15)	−0.0041 (14)	−0.0017 (14)
C12B	0.0340 (19)	0.0199 (16)	0.0250 (15)	0.0016 (14)	0.0025 (13)	0.0006 (12)
C13B	0.0258 (17)	0.0226 (17)	0.0290 (16)	0.0022 (13)	0.0033 (13)	0.0008 (13)
C14B	0.0291 (17)	0.0204 (16)	0.0269 (15)	0.0040 (13)	0.0054 (13)	−0.0016 (12)
C15B	0.0244 (17)	0.0270 (18)	0.0245 (15)	−0.0012 (13)	−0.0006 (12)	−0.0011 (13)
C16B	0.039 (2)	0.0299 (19)	0.0255 (16)	0.0057 (16)	0.0045 (14)	−0.0062 (14)
C17B	0.039 (2)	0.038 (2)	0.0214 (15)	0.0036 (16)	−0.0011 (14)	−0.0004 (14)
C18B	0.0264 (17)	0.0289 (18)	0.0249 (15)	0.0014 (14)	−0.0031 (13)	0.0024 (13)
C19B	0.0243 (17)	0.0253 (17)	0.0229 (14)	0.0012 (13)	−0.0008 (12)	0.0008 (12)
C20B	0.0252 (17)	0.0290 (18)	0.0181 (14)	0.0043 (13)	0.0005 (12)	0.0009 (12)
C21B	0.0258 (17)	0.0240 (17)	0.0208 (14)	0.0016 (13)	−0.0001 (12)	−0.0008 (12)
C22B	0.0210 (16)	0.0240 (17)	0.0246 (15)	0.0065 (13)	−0.0005 (12)	−0.0012 (12)
C23B	0.0275 (18)	0.0266 (18)	0.0297 (16)	0.0028 (14)	−0.0055 (13)	−0.0018 (13)
C24B	0.0217 (17)	0.0324 (19)	0.0327 (17)	0.0006 (14)	0.0020 (13)	0.0008 (14)
C25B	0.0291 (18)	0.0289 (18)	0.0269 (16)	0.0061 (14)	0.0061 (13)	0.0043 (13)
C26B	0.0251 (17)	0.0212 (16)	0.0237 (14)	0.0027 (13)	−0.0019 (12)	0.0008 (12)
C27B	0.0202 (16)	0.0229 (17)	0.0283 (15)	0.0050 (13)	−0.0010 (12)	−0.0023 (12)
C28B	0.0312 (18)	0.0248 (17)	0.0207 (14)	0.0029 (14)	0.0032 (12)	−0.0004 (12)
C29B	0.078 (3)	0.063 (3)	0.0216 (17)	0.023 (3)	0.0114 (19)	−0.0030 (18)
C30B	0.049 (2)	0.042 (2)	0.0313 (18)	0.0049 (19)	0.0023 (16)	0.0118 (16)
C31B	0.056 (3)	0.037 (2)	0.037 (2)	−0.0040 (19)	0.0027 (18)	0.0060 (17)
C32B	0.062 (4)	0.138 (7)	0.071 (4)	−0.001 (4)	0.020 (3)	−0.021 (4)
C33B	0.0286 (18)	0.0279 (18)	0.0341 (17)	0.0029 (14)	−0.0032 (14)	0.0055 (14)
C34B	0.034 (2)	0.043 (2)	0.0364 (19)	0.0056 (17)	−0.0036 (15)	0.0059 (16)
C35B	0.0321 (19)	0.036 (2)	0.0338 (18)	−0.0001 (16)	−0.0053 (14)	0.0072 (15)
C36B	0.038 (2)	0.033 (2)	0.075 (3)	0.0004 (18)	−0.007 (2)	−0.008 (2)
C37B	0.042 (2)	0.039 (2)	0.070 (3)	0.0081 (19)	−0.003 (2)	−0.008 (2)
C38B	0.032 (2)	0.051 (3)	0.053 (2)	−0.0052 (19)	−0.0086 (18)	0.002 (2)
C39B	0.050 (3)	0.047 (3)	0.063 (3)	−0.008 (2)	−0.016 (2)	−0.008 (2)
C40B	0.044 (2)	0.044 (2)	0.050 (2)	0.0033 (19)	−0.0125 (19)	−0.0105 (19)
C41B	0.036 (2)	0.076 (3)	0.038 (2)	−0.008 (2)	0.0077 (17)	0.001 (2)
C42B	0.034 (2)	0.073 (3)	0.045 (2)	0.007 (2)	−0.0016 (18)	−0.011 (2)
C43B	0.055 (3)	0.073 (4)	0.048 (3)	−0.002 (3)	−0.010 (2)	−0.002 (2)
C44B	0.216 (10)	0.112 (6)	0.036 (3)	0.008 (6)	−0.010 (4)	−0.014 (3)
C45B	0.0309 (19)	0.0288 (18)	0.0307 (17)	0.0034 (15)	0.0067 (14)	0.0043 (14)
C46B	0.032 (2)	0.031 (2)	0.0394 (19)	0.0048 (16)	0.0057 (15)	0.0060 (16)
C47B	0.0290 (19)	0.038 (2)	0.0408 (19)	0.0105 (16)	0.0051 (15)	0.0120 (16)
C48B	0.044 (3)	0.066 (3)	0.046 (2)	0.006 (2)	0.0087 (19)	0.002 (2)
C49B	0.037 (2)	0.091 (4)	0.061 (3)	0.006 (3)	0.019 (2)	0.008 (3)
C50B	0.039 (3)	0.066 (3)	0.071 (3)	−0.008 (2)	0.000 (2)	0.012 (3)
C51B	0.057 (3)	0.051 (3)	0.066 (3)	0.002 (2)	−0.008 (2)	0.001 (2)
C52B	0.043 (2)	0.044 (2)	0.055 (2)	0.010 (2)	0.0054 (19)	−0.003 (2)
C53B	0.207 (8)	0.082 (4)	0.033 (2)	0.079 (5)	0.019 (3)	−0.009 (3)
C54B	0.048 (2)	0.042 (2)	0.0324 (19)	0.0068 (19)	−0.0085 (17)	0.0043 (17)
C55B	0.059 (3)	0.052 (3)	0.053 (3)	0.002 (2)	−0.024 (2)	−0.002 (2)
C56B	0.082 (5)	0.120 (7)	0.207 (10)	−0.015 (5)	−0.085 (6)	−0.030 (7)
C57B	0.0229 (17)	0.0294 (18)	0.0253 (15)	0.0029 (14)	0.0027 (12)	0.0003 (13)
C58B	0.0287 (18)	0.0253 (18)	0.0317 (17)	0.0002 (14)	−0.0011 (14)	0.0003 (14)
C59B	0.0292 (19)	0.0248 (18)	0.0327 (17)	−0.0002 (14)	0.0003 (14)	−0.0031 (14)
C60B	0.038 (2)	0.042 (2)	0.0345 (19)	−0.0067 (18)	−0.0009 (16)	−0.0024 (16)
C61B	0.038 (2)	0.047 (3)	0.049 (2)	−0.0115 (19)	0.0128 (18)	−0.0028 (19)
C62B	0.035 (2)	0.042 (2)	0.060 (3)	−0.0083 (19)	−0.0028 (19)	−0.008 (2)
C63B	0.043 (2)	0.054 (3)	0.042 (2)	−0.009 (2)	−0.0055 (18)	−0.0066 (19)
C64B	0.037 (2)	0.048 (2)	0.0315 (18)	−0.0095 (18)	0.0041 (15)	−0.0007 (16)
C65B	0.027 (2)	0.064 (3)	0.045 (2)	−0.0070 (19)	−0.0034 (17)	−0.008 (2)
C66B	0.034 (2)	0.033 (2)	0.0377 (19)	−0.0012 (16)	0.0069 (15)	0.0074 (15)
C67B	0.034 (2)	0.040 (2)	0.039 (2)	−0.0075 (18)	0.0011 (16)	0.0032 (17)
C68B	0.082 (4)	0.059 (3)	0.049 (3)	−0.005 (3)	0.032 (2)	−0.007 (2)
C69B	0.0344 (19)	0.0293 (18)	0.0233 (15)	−0.0070 (15)	0.0028 (13)	−0.0023 (13)
C70B	0.062 (3)	0.050 (3)	0.0260 (18)	−0.027 (2)	0.0005 (17)	−0.0021 (17)
C71B	0.051 (3)	0.058 (3)	0.0240 (17)	−0.021 (2)	0.0019 (16)	−0.0103 (17)
C72B	0.062 (3)	0.058 (3)	0.048 (2)	−0.024 (2)	−0.009 (2)	0.008 (2)
C73B	0.077 (4)	0.075 (4)	0.068 (3)	−0.036 (3)	−0.009 (3)	0.014 (3)
C74B	0.068 (4)	0.100 (5)	0.057 (3)	−0.050 (4)	0.002 (3)	0.003 (3)
C75B	0.041 (3)	0.119 (5)	0.040 (2)	−0.006 (3)	0.0136 (19)	−0.019 (3)
C76B	0.064 (3)	0.067 (3)	0.040 (2)	−0.003 (3)	0.011 (2)	−0.019 (2)
O1C	0.0572 (18)	0.0312 (14)	0.0275 (12)	−0.0055 (13)	0.0042 (11)	0.0070 (10)
O2C	0.0383 (15)	0.0328 (14)	0.0307 (12)	−0.0051 (11)	−0.0053 (10)	−0.0027 (10)
O3C	0.080 (3)	0.100 (3)	0.057 (2)	−0.018 (2)	−0.0243 (19)	0.039 (2)
O4C	0.056 (2)	0.059 (2)	0.077 (2)	0.0070 (17)	−0.0271 (18)	0.0057 (18)
O5C	0.0256 (13)	0.0475 (16)	0.0311 (12)	0.0027 (11)	−0.0069 (10)	0.0063 (11)
O6C	0.0301 (13)	0.0284 (13)	0.0332 (12)	−0.0001 (10)	0.0062 (10)	−0.0004 (10)
O7C	0.060 (2)	0.070 (2)	0.067 (2)	0.0127 (18)	−0.0038 (16)	−0.0343 (19)
O8C	0.095 (3)	0.049 (2)	0.0506 (18)	0.0105 (19)	0.0369 (18)	0.0010 (15)
O9C	0.0628 (19)	0.0354 (15)	0.0214 (12)	−0.0171 (14)	0.0027 (12)	0.0041 (10)
C54C	0.083 (4)	0.043 (3)	0.066 (3)	−0.004 (3)	0.019 (3)	−0.012 (2)
O11C	0.174 (6)	0.236 (9)	0.093 (4)	−0.003 (6)	0.066 (4)	−0.024 (5)
O12C	0.113 (4)	0.074 (3)	0.074 (3)	0.001 (3)	−0.023 (2)	−0.020 (2)
O13C	0.0360 (15)	0.0466 (17)	0.0351 (13)	0.0047 (13)	0.0113 (11)	−0.0010 (12)
O14C	0.0296 (14)	0.0410 (16)	0.0360 (13)	−0.0049 (12)	−0.0040 (10)	0.0078 (11)
O15C	0.100 (3)	0.148 (5)	0.062 (2)	−0.074 (3)	−0.025 (2)	0.009 (3)
O16C	0.0499 (18)	0.066 (2)	0.0401 (15)	−0.0125 (16)	−0.0094 (13)	−0.0007 (14)
C1C	0.0264 (17)	0.0260 (17)	0.0273 (16)	0.0004 (14)	0.0042 (13)	0.0008 (13)
C2C	0.0296 (18)	0.0289 (18)	0.0275 (16)	−0.0032 (14)	0.0037 (13)	0.0049 (13)
C3C	0.033 (2)	0.036 (2)	0.0262 (16)	−0.0006 (16)	0.0004 (14)	0.0037 (14)
C4C	0.0271 (18)	0.0296 (18)	0.0268 (16)	−0.0032 (14)	0.0004 (13)	0.0005 (13)
C5C	0.0217 (16)	0.0249 (17)	0.0258 (15)	−0.0005 (13)	0.0010 (12)	0.0043 (12)
C6C	0.0220 (16)	0.0304 (18)	0.0243 (15)	−0.0021 (13)	−0.0002 (12)	0.0032 (13)
C7C	0.0245 (17)	0.0264 (17)	0.0234 (15)	−0.0051 (13)	−0.0020 (12)	0.0046 (13)
C8C	0.0271 (17)	0.0213 (16)	0.0262 (15)	−0.0043 (13)	−0.0022 (13)	0.0049 (12)
C9C	0.0253 (17)	0.0267 (18)	0.0279 (16)	−0.0032 (14)	−0.0043 (13)	0.0049 (13)
C10C	0.0230 (17)	0.0271 (18)	0.0344 (17)	−0.0030 (14)	−0.0011 (13)	0.0023 (14)
C11C	0.0265 (17)	0.0229 (17)	0.0297 (16)	−0.0048 (14)	0.0039 (13)	0.0025 (13)
C12C	0.0253 (16)	0.0193 (16)	0.0263 (15)	−0.0042 (13)	−0.0024 (12)	0.0038 (12)
C13C	0.0183 (15)	0.0239 (17)	0.0290 (15)	−0.0022 (13)	−0.0019 (12)	0.0041 (13)
C14C	0.0302 (18)	0.0232 (17)	0.0230 (14)	−0.0017 (14)	0.0030 (12)	0.0039 (12)
C15C	0.0268 (17)	0.0265 (18)	0.0272 (16)	−0.0008 (14)	−0.0029 (13)	0.0011 (13)
C16C	0.0312 (19)	0.0306 (19)	0.0263 (16)	−0.0070 (15)	0.0002 (13)	0.0012 (14)
C17C	0.045 (2)	0.037 (2)	0.0232 (16)	−0.0068 (17)	0.0013 (14)	−0.0013 (14)
C18C	0.043 (2)	0.0281 (19)	0.0284 (17)	−0.0066 (16)	0.0000 (15)	−0.0040 (14)
C19C	0.0318 (19)	0.0278 (18)	0.0261 (16)	−0.0063 (15)	−0.0014 (13)	−0.0003 (13)
C20C	0.0276 (18)	0.0296 (18)	0.0229 (15)	−0.0007 (14)	0.0029 (13)	0.0014 (13)
C21C	0.039 (2)	0.0220 (17)	0.0262 (16)	−0.0044 (15)	0.0030 (14)	−0.0016 (13)
C22C	0.0317 (18)	0.0188 (16)	0.0323 (17)	−0.0022 (14)	0.0027 (14)	0.0007 (13)
C23C	0.0314 (19)	0.0246 (17)	0.0346 (17)	−0.0001 (14)	0.0076 (14)	0.0009 (14)
C24C	0.0250 (18)	0.0311 (19)	0.0403 (19)	0.0030 (15)	0.0025 (14)	0.0021 (15)
C25C	0.0308 (19)	0.0270 (18)	0.0309 (17)	0.0015 (15)	−0.0015 (14)	0.0029 (14)
C26C	0.0271 (17)	0.0209 (16)	0.0279 (15)	−0.0036 (13)	0.0043 (13)	0.0027 (12)
C27C	0.0302 (18)	0.0203 (17)	0.0310 (16)	−0.0016 (14)	0.0008 (13)	0.0021 (13)
C28C	0.0312 (18)	0.0232 (17)	0.0275 (16)	−0.0035 (14)	0.0049 (13)	0.0052 (13)
C29C	0.087 (4)	0.055 (3)	0.033 (2)	−0.010 (3)	0.000 (2)	0.011 (2)
C30C	0.037 (2)	0.035 (2)	0.041 (2)	−0.0070 (17)	−0.0123 (16)	0.0038 (16)
C31C	0.059 (3)	0.041 (2)	0.046 (2)	−0.010 (2)	−0.026 (2)	0.0041 (19)
C32C	0.088 (5)	0.083 (5)	0.137 (6)	0.015 (4)	−0.067 (5)	0.019 (4)
C33C	0.0271 (18)	0.0292 (19)	0.0294 (16)	−0.0023 (14)	0.0014 (13)	0.0042 (14)
C34C	0.0303 (19)	0.0268 (18)	0.0364 (18)	−0.0017 (15)	−0.0018 (14)	0.0052 (14)
C35C	0.0318 (19)	0.0267 (19)	0.0358 (18)	0.0011 (15)	−0.0009 (15)	0.0068 (15)
C36C	0.062 (3)	0.058 (3)	0.039 (2)	0.024 (2)	−0.002 (2)	−0.001 (2)
C37C	0.070 (3)	0.064 (3)	0.056 (3)	0.035 (3)	0.019 (2)	0.003 (2)
C38C	0.038 (2)	0.051 (3)	0.071 (3)	0.014 (2)	0.010 (2)	0.020 (2)
C39C	0.042 (3)	0.050 (3)	0.061 (3)	0.003 (2)	−0.016 (2)	0.007 (2)
C40C	0.036 (2)	0.050 (3)	0.044 (2)	0.0037 (19)	−0.0004 (17)	−0.0061 (18)
C41C	0.033 (2)	0.056 (3)	0.047 (2)	0.0044 (19)	−0.0050 (17)	0.008 (2)
C42C	0.034 (2)	0.0294 (19)	0.044 (2)	0.0025 (16)	0.0022 (16)	−0.0036 (16)
C43C	0.040 (2)	0.047 (3)	0.046 (2)	0.0157 (19)	−0.0042 (18)	−0.008 (2)
C44C	0.118 (6)	0.089 (5)	0.061 (3)	0.037 (4)	0.043 (3)	0.013 (3)
C45C	0.0332 (19)	0.033 (2)	0.0300 (17)	0.0035 (15)	−0.0012 (14)	0.0054 (14)
C46C	0.049 (3)	0.059 (3)	0.0286 (18)	0.021 (2)	−0.0040 (16)	0.0050 (18)
C47C	0.050 (3)	0.061 (3)	0.0244 (17)	0.017 (2)	−0.0015 (16)	0.0110 (17)
C48C	0.049 (3)	0.057 (3)	0.056 (3)	0.016 (2)	−0.009 (2)	0.000 (2)
C49C	0.059 (3)	0.066 (4)	0.069 (3)	0.025 (3)	−0.004 (3)	−0.006 (3)
C50C	0.047 (3)	0.083 (4)	0.060 (3)	0.025 (3)	0.004 (2)	0.010 (3)
C51C	0.040 (2)	0.081 (4)	0.037 (2)	0.004 (2)	0.0116 (17)	0.013 (2)
C52C	0.054 (3)	0.063 (3)	0.038 (2)	0.006 (2)	0.0077 (19)	0.016 (2)
C53C	0.128 (5)	0.068 (3)	0.0209 (19)	−0.056 (4)	0.007 (2)	−0.001 (2)
O10C	0.077 (2)	0.0334 (15)	0.0348 (14)	−0.0098 (15)	0.0110 (13)	−0.0101 (12)
C55C	0.095 (5)	0.073 (4)	0.065 (3)	0.001 (4)	0.028 (3)	−0.016 (3)
C56C	0.162 (8)	0.145 (8)	0.074 (5)	−0.031 (7)	−0.012 (5)	−0.021 (5)
C57C	0.036 (2)	0.0266 (18)	0.0334 (17)	−0.0039 (15)	−0.0011 (14)	0.0003 (14)
C58C	0.049 (2)	0.036 (2)	0.0380 (19)	−0.0118 (18)	−0.0099 (17)	0.0018 (16)
C59C	0.055 (3)	0.031 (2)	0.051 (2)	−0.0112 (19)	−0.0189 (19)	−0.0037 (18)
C60C	0.040 (3)	0.044 (3)	0.090 (4)	−0.002 (2)	−0.019 (2)	0.018 (3)
C61C	0.050 (3)	0.045 (3)	0.115 (5)	−0.004 (2)	−0.017 (3)	0.008 (3)
C62C	0.056 (3)	0.054 (3)	0.132 (6)	0.004 (3)	−0.041 (4)	−0.022 (4)
C63C	0.090 (5)	0.077 (4)	0.074 (4)	0.025 (4)	−0.051 (3)	−0.016 (3)
C64C	0.083 (4)	0.062 (3)	0.050 (3)	−0.003 (3)	−0.031 (2)	0.000 (2)
C65C	0.036 (2)	0.066 (3)	0.054 (2)	0.014 (2)	0.0203 (19)	0.005 (2)
C66C	0.064 (3)	0.129 (6)	0.045 (3)	−0.051 (4)	−0.012 (2)	0.016 (3)
C67C	0.050 (3)	0.092 (4)	0.044 (2)	−0.029 (3)	−0.008 (2)	0.000 (2)
C68C	0.083 (4)	0.081 (4)	0.035 (2)	−0.004 (3)	−0.012 (2)	0.001 (2)
C69C	0.0282 (18)	0.0283 (18)	0.0371 (18)	−0.0046 (15)	0.0087 (14)	−0.0032 (15)
C70C	0.032 (2)	0.0272 (19)	0.047 (2)	−0.0064 (16)	0.0067 (16)	−0.0055 (16)
C71C	0.033 (2)	0.031 (2)	0.071 (3)	−0.0090 (17)	0.0081 (19)	−0.010 (2)
C72C	0.038 (3)	0.051 (3)	0.113 (4)	−0.011 (2)	−0.007 (3)	0.018 (3)
C73C	0.048 (4)	0.059 (4)	0.221 (9)	−0.008 (3)	−0.034 (5)	0.037 (5)
C74C	0.031 (3)	0.065 (5)	0.301 (14)	−0.002 (3)	0.023 (6)	−0.009 (7)
C75C	0.052 (4)	0.089 (5)	0.188 (8)	−0.013 (4)	0.060 (5)	−0.021 (6)
C76C	0.047 (3)	0.067 (3)	0.091 (4)	−0.019 (3)	0.026 (3)	−0.011 (3)
O1D	0.0489 (16)	0.0317 (14)	0.0235 (11)	0.0008 (12)	0.0046 (10)	−0.0070 (10)
O2D	0.0319 (14)	0.0323 (14)	0.0299 (12)	0.0030 (11)	−0.0024 (10)	0.0057 (10)
O3D	0.072 (2)	0.092 (3)	0.0454 (18)	0.014 (2)	−0.0192 (17)	−0.0190 (19)
O4D	0.048 (2)	0.062 (2)	0.070 (2)	−0.0097 (16)	−0.0229 (16)	−0.0108 (17)
O5D	0.0229 (12)	0.0432 (15)	0.0266 (11)	0.0001 (11)	−0.0054 (9)	−0.0046 (10)
O6D	0.0314 (13)	0.0247 (12)	0.0291 (12)	−0.0022 (10)	0.0083 (10)	−0.0040 (9)
O7D	0.066 (2)	0.0456 (19)	0.0582 (19)	−0.0045 (16)	0.0135 (16)	0.0159 (15)
O8D	0.068 (2)	0.0372 (16)	0.0415 (15)	−0.0041 (15)	0.0246 (14)	−0.0015 (12)
O9D	0.068 (2)	0.0470 (18)	0.0229 (13)	0.0121 (15)	0.0080 (12)	−0.0029 (12)
O10D	0.066 (2)	0.0449 (17)	0.0290 (13)	−0.0021 (15)	0.0100 (12)	0.0124 (12)
O11D	0.129 (4)	0.209 (7)	0.051 (2)	−0.071 (4)	0.021 (2)	0.029 (3)
O12D	0.073 (3)	0.113 (3)	0.0442 (18)	−0.001 (2)	−0.0042 (17)	0.015 (2)
O13D	0.0304 (13)	0.0445 (15)	0.0282 (11)	−0.0058 (11)	0.0070 (10)	0.0016 (11)
O14D	0.0252 (12)	0.0397 (14)	0.0230 (10)	−0.0041 (11)	−0.0026 (9)	−0.0024 (10)
O15D	0.058 (2)	0.146 (4)	0.0466 (18)	0.047 (2)	−0.0134 (15)	0.001 (2)
O16D	0.0404 (15)	0.0494 (17)	0.0273 (12)	0.0010 (12)	−0.0043 (11)	0.0002 (11)
C1D	0.0221 (16)	0.0267 (17)	0.0237 (15)	−0.0009 (13)	0.0016 (12)	0.0013 (13)
C2D	0.0270 (17)	0.0270 (17)	0.0229 (15)	−0.0047 (14)	0.0023 (12)	−0.0043 (13)
C3D	0.0300 (18)	0.035 (2)	0.0195 (14)	−0.0033 (15)	0.0002 (12)	−0.0009 (13)
C4D	0.0241 (17)	0.0290 (18)	0.0243 (15)	0.0009 (14)	−0.0033 (13)	0.0034 (13)
C5D	0.0214 (16)	0.0286 (18)	0.0232 (15)	0.0005 (14)	−0.0016 (12)	−0.0041 (13)
C6D	0.0239 (16)	0.0265 (17)	0.0211 (14)	−0.0002 (13)	0.0005 (12)	−0.0012 (12)
C7D	0.0233 (16)	0.0225 (16)	0.0246 (15)	0.0000 (13)	−0.0001 (12)	−0.0008 (12)
C8D	0.0224 (16)	0.0231 (16)	0.0221 (14)	0.0048 (13)	0.0009 (12)	−0.0028 (12)
C9D	0.0261 (17)	0.0245 (17)	0.0261 (15)	0.0045 (13)	−0.0028 (13)	−0.0040 (12)
C10D	0.0200 (16)	0.0292 (18)	0.0305 (16)	0.0040 (14)	−0.0009 (13)	−0.0034 (14)
C11D	0.0268 (17)	0.0197 (16)	0.0283 (16)	0.0042 (13)	0.0017 (13)	−0.0026 (12)
C12D	0.0268 (17)	0.0228 (16)	0.0243 (15)	0.0026 (13)	−0.0024 (12)	−0.0055 (12)
C13D	0.0219 (16)	0.0238 (17)	0.0270 (15)	0.0026 (13)	−0.0016 (12)	−0.0028 (12)
C14D	0.0258 (17)	0.0319 (19)	0.0230 (15)	0.0014 (14)	−0.0027 (12)	−0.0063 (13)
C15D	0.0230 (17)	0.037 (2)	0.0208 (14)	−0.0003 (14)	−0.0047 (12)	−0.0010 (13)
C16D	0.035 (2)	0.042 (2)	0.0217 (15)	0.0047 (16)	−0.0028 (14)	−0.0036 (14)
C17D	0.048 (2)	0.047 (2)	0.0184 (15)	0.0031 (18)	0.0027 (15)	0.0019 (15)
C18D	0.039 (2)	0.041 (2)	0.0265 (17)	−0.0034 (17)	−0.0027 (15)	0.0056 (15)
C19D	0.0260 (17)	0.0311 (19)	0.0254 (15)	−0.0019 (14)	−0.0033 (13)	−0.0017 (13)
C20D	0.0233 (17)	0.0368 (19)	0.0183 (14)	−0.0016 (14)	0.0002 (12)	−0.0001 (13)
C21D	0.0356 (19)	0.0293 (19)	0.0223 (15)	−0.0037 (15)	−0.0031 (13)	0.0020 (13)
C22D	0.0327 (18)	0.0239 (17)	0.0220 (14)	−0.0018 (14)	−0.0016 (13)	0.0029 (12)
C23D	0.0323 (18)	0.0238 (17)	0.0258 (15)	−0.0012 (14)	0.0070 (13)	0.0009 (13)
C24D	0.0238 (17)	0.0281 (18)	0.0285 (15)	−0.0029 (14)	−0.0008 (13)	−0.0016 (14)
C25D	0.0265 (17)	0.0207 (16)	0.0248 (15)	0.0012 (13)	0.0007 (12)	−0.0010 (12)
C26D	0.0277 (18)	0.0223 (17)	0.0223 (14)	−0.0032 (13)	0.0015 (12)	0.0006 (12)
C27D	0.0234 (17)	0.0226 (17)	0.0296 (16)	−0.0018 (13)	0.0007 (13)	0.0012 (13)
C28D	0.0268 (17)	0.0235 (17)	0.0237 (15)	0.0009 (13)	0.0036 (12)	0.0004 (12)
C29D	0.060 (3)	0.043 (2)	0.0259 (17)	0.003 (2)	−0.0002 (17)	−0.0114 (16)
C30D	0.034 (2)	0.038 (2)	0.0341 (18)	0.0073 (16)	−0.0109 (15)	0.0002 (15)
C31D	0.049 (3)	0.035 (2)	0.044 (2)	0.0111 (19)	−0.0204 (19)	−0.0037 (17)
C32D	0.093 (5)	0.069 (4)	0.109 (5)	−0.023 (3)	−0.054 (4)	−0.012 (4)
C33D	0.0239 (17)	0.028 (2)	0.0289 (17)	0.0025 (14)	0.0026 (13)	−0.0038 (13)
C34D	0.0234 (17)	0.033 (2)	0.0349 (17)	0.0020 (14)	−0.0003 (14)	−0.0050 (15)
C35D	0.031 (2)	0.0220 (18)	0.0370 (18)	−0.0012 (15)	0.0045 (15)	−0.0025 (14)
C36D	0.047 (2)	0.040 (2)	0.039 (2)	−0.0071 (18)	0.0044 (17)	−0.0002 (17)
C37D	0.056 (3)	0.042 (2)	0.057 (3)	−0.020 (2)	0.016 (2)	−0.004 (2)
C38D	0.032 (2)	0.044 (2)	0.069 (3)	−0.0080 (18)	0.005 (2)	−0.019 (2)
C39D	0.035 (2)	0.058 (3)	0.050 (2)	0.003 (2)	−0.0036 (19)	−0.007 (2)
C40D	0.037 (2)	0.045 (2)	0.0376 (19)	−0.0045 (18)	−0.0005 (16)	0.0009 (17)
C41D	0.035 (2)	0.055 (3)	0.037 (2)	−0.0089 (18)	−0.0090 (16)	−0.0075 (18)
C42D	0.033 (2)	0.0240 (18)	0.0389 (19)	−0.0015 (15)	−0.0015 (15)	0.0012 (14)
C43D	0.042 (2)	0.036 (2)	0.0335 (18)	−0.0058 (17)	0.0001 (16)	0.0018 (16)
C44D	0.098 (4)	0.063 (3)	0.051 (3)	−0.007 (3)	0.046 (3)	−0.003 (2)
C45D	0.0308 (19)	0.036 (2)	0.0248 (16)	−0.0055 (15)	−0.0026 (13)	−0.0015 (14)
C46D	0.047 (3)	0.064 (3)	0.0298 (19)	−0.022 (2)	−0.0068 (16)	−0.0006 (18)
C47D	0.047 (3)	0.066 (3)	0.0231 (17)	−0.019 (2)	0.0017 (16)	−0.0126 (18)
C48D	0.050 (3)	0.070 (3)	0.044 (2)	−0.014 (2)	−0.006 (2)	0.001 (2)
C49D	0.061 (3)	0.068 (4)	0.063 (3)	−0.026 (3)	−0.003 (2)	0.007 (3)
C50D	0.051 (3)	0.088 (4)	0.046 (3)	−0.028 (3)	0.005 (2)	−0.007 (3)
C51D	0.038 (2)	0.091 (4)	0.035 (2)	−0.016 (2)	0.0099 (17)	−0.019 (2)
C52D	0.051 (3)	0.067 (3)	0.032 (2)	−0.005 (2)	0.0058 (18)	−0.0172 (19)
C53D	0.115 (5)	0.083 (4)	0.0167 (18)	0.048 (3)	0.010 (2)	−0.002 (2)
C54D	0.066 (3)	0.056 (3)	0.052 (3)	−0.018 (3)	0.002 (2)	0.012 (2)
C55D	0.079 (4)	0.057 (3)	0.043 (2)	−0.010 (3)	0.013 (2)	0.013 (2)
C56D	0.133 (7)	0.141 (7)	0.054 (3)	0.009 (5)	−0.038 (4)	−0.003 (4)
C57D	0.035 (2)	0.036 (2)	0.0270 (16)	0.0012 (16)	−0.0061 (14)	0.0039 (14)
C58D	0.039 (2)	0.078 (3)	0.039 (2)	0.000 (2)	−0.0046 (17)	0.018 (2)
C59D	0.049 (2)	0.046 (2)	0.0364 (19)	0.0013 (19)	−0.0108 (17)	0.0086 (17)
C60D	0.056 (3)	0.077 (4)	0.084 (4)	0.013 (3)	−0.028 (3)	−0.038 (3)
C61D	0.058 (4)	0.143 (7)	0.096 (5)	0.032 (4)	−0.011 (3)	−0.041 (5)
C62D	0.047 (3)	0.118 (6)	0.065 (3)	−0.014 (3)	−0.017 (2)	0.003 (3)
C63D	0.087 (4)	0.076 (4)	0.064 (3)	−0.024 (3)	−0.038 (3)	0.002 (3)
C64D	0.069 (3)	0.073 (4)	0.043 (2)	0.010 (3)	−0.018 (2)	−0.010 (2)
C65D	0.037 (2)	0.037 (2)	0.0407 (19)	−0.0032 (17)	0.0104 (16)	−0.0077 (16)
C66D	0.035 (2)	0.062 (3)	0.0344 (18)	0.0153 (19)	−0.0014 (15)	−0.0046 (18)
C67D	0.042 (2)	0.071 (3)	0.0325 (19)	0.010 (2)	−0.0041 (17)	0.0015 (19)
C68D	0.059 (3)	0.058 (3)	0.034 (2)	−0.007 (2)	−0.0142 (19)	0.0001 (19)
C69D	0.0284 (18)	0.0288 (18)	0.0337 (17)	−0.0024 (15)	0.0088 (14)	0.0054 (14)
C70D	0.036 (2)	0.036 (2)	0.046 (2)	0.0039 (17)	0.0048 (17)	0.0035 (17)
C71D	0.029 (2)	0.033 (2)	0.075 (3)	0.0055 (17)	0.0044 (19)	0.011 (2)
C72D	0.046 (3)	0.093 (5)	0.094 (4)	0.018 (3)	0.025 (3)	0.043 (4)
C73D	0.046 (4)	0.113 (7)	0.185 (9)	0.029 (4)	0.048 (5)	0.078 (7)
C74D	0.047 (4)	0.083 (6)	0.256 (13)	−0.013 (4)	0.005 (6)	0.038 (7)
C75D	0.047 (3)	0.058 (4)	0.207 (9)	−0.004 (3)	−0.024 (5)	−0.017 (5)
C76D	0.037 (3)	0.052 (3)	0.118 (5)	0.005 (2)	−0.005 (3)	−0.020 (3)

### Geometric parameters (Å, °)

**Table:**

O1A—C2A	1.373 (4)	O1C—C2C	1.381 (4)
O1A—C29A	1.430 (5)	O1C—C29C	1.439 (5)
O2A—C4A	1.398 (4)	O2C—C4C	1.394 (4)
O2A—C30A	1.425 (5)	O2C—C30C	1.431 (5)
O3A—C31A	1.196 (6)	O3C—C31C	1.177 (6)
O4A—C31A	1.341 (6)	O4C—C31C	1.354 (6)
O4A—C32A	1.452 (7)	O4C—C32C	1.454 (6)
O5A—C9A	1.375 (4)	O5C—C9C	1.375 (4)
O5A—C41A	1.412 (4)	O5C—C41C	1.433 (5)
O6A—C11A	1.393 (4)	O6C—C11C	1.388 (4)
O6A—C42A	1.415 (4)	O6C—C42C	1.425 (5)
O7A—C43A	1.209 (5)	O7C—C43C	1.201 (5)
O8A—C43A	1.316 (5)	O8C—C43C	1.333 (6)
O8A—C44A	1.443 (5)	O8C—C44C	1.458 (6)
O9A—C16A	1.362 (4)	O9C—C16C	1.374 (4)
O9A—C53A	1.412 (4)	O9C—C53C	1.403 (5)
O10A—C18A	1.383 (5)	C54C—C55C	1.428 (8)
O10A—C54A	1.426 (4)	C54C—O10C	1.444 (6)
O11A—C55A	1.197 (6)	C54C—H54E	0.9900
O12A—C55A	1.289 (5)	C54C—H54F	0.9900
O12A—C56A	1.454 (6)	O11C—C55C	1.166 (7)
O13A—C23A	1.375 (4)	O12C—C55C	1.338 (8)
O13A—C65A	1.426 (5)	O12C—C56C	1.536 (8)
O14A—C25A	1.382 (4)	O13C—C23C	1.373 (4)
O14A—C66A	1.396 (5)	O13C—C65C	1.427 (5)
O15A—C67A	1.196 (6)	O14C—C25C	1.399 (4)
O16A—C67A	1.314 (5)	O14C—C66C	1.402 (6)
O16A—C68A	1.441 (5)	O15C—C67C	1.193 (6)
C1A—C6A	1.393 (5)	O16C—C67C	1.337 (6)
C1A—C2A	1.404 (4)	O16C—C68C	1.455 (5)
C1A—C28A	1.529 (5)	C1C—C6C	1.393 (5)
C2A—C3A	1.377 (5)	C1C—C2C	1.403 (5)
C3A—C4A	1.392 (5)	C1C—C28C	1.517 (5)
C3A—H3A	0.9500	C2C—C3C	1.372 (5)
C4A—C5A	1.395 (4)	C3C—C4C	1.390 (5)
C5A—C6A	1.393 (5)	C3C—H3C	0.9500
C5A—C7A	1.524 (5)	C4C—C5C	1.397 (4)
C6A—H6A	0.9500	C5C—C6C	1.382 (5)
C7A—C8A	1.528 (4)	C5C—C7C	1.534 (4)
C7A—C33A	1.553 (5)	C6C—H6C	0.9500
C7A—H7A	1.0000	C7C—C8C	1.518 (5)
C8A—C13A	1.393 (4)	C7C—C33C	1.532 (5)
C8A—C9A	1.401 (5)	C7C—H7C	1.0000
C9A—C10A	1.391 (5)	C8C—C13C	1.388 (4)
C10A—C11A	1.388 (5)	C8C—C9C	1.405 (5)
C10A—H10A	0.9500	C9C—C10C	1.381 (5)
C11A—C12A	1.390 (5)	C10C—C11C	1.388 (5)
C12A—C13A	1.409 (4)	C10C—H10C	0.9500
C12A—C14A	1.511 (4)	C11C—C12C	1.389 (5)
C13A—H13A	0.9500	C12C—C13C	1.398 (5)
C14A—C15A	1.513 (5)	C12C—C14C	1.516 (4)
C14A—C45A	1.546 (5)	C13C—H13C	0.9500
C14A—H14A	1.0000	C14C—C15C	1.514 (5)
C15A—C20A	1.397 (5)	C14C—C45C	1.535 (5)
C15A—C16A	1.400 (4)	C14C—H14C	1.0000
C16A—C17A	1.393 (5)	C15C—C16C	1.396 (5)
C17A—C18A	1.387 (5)	C15C—C20C	1.397 (5)
C17A—H17A	0.9500	C16C—C17C	1.381 (5)
C18A—C19A	1.405 (4)	C17C—C18C	1.393 (5)
C19A—C20A	1.384 (5)	C17C—H17C	0.9500
C19A—C21A	1.518 (5)	C18C—O10C	1.387 (5)
C20A—H20A	0.9500	C18C—C19C	1.395 (5)
C21A—C57A	1.531 (5)	C19C—C20C	1.388 (5)
C21A—C22A	1.534 (5)	C19C—C21C	1.519 (5)
C21A—H21A	1.0000	C20C—H20C	0.9500
C22A—C27A	1.393 (4)	C21C—C22C	1.519 (5)
C22A—C23A	1.397 (5)	C21C—C57C	1.528 (5)
C23A—C24A	1.395 (5)	C21C—H21C	1.0000
C24A—C25A	1.383 (5)	C22C—C27C	1.394 (5)
C24A—H24A	0.9500	C22C—C23C	1.402 (5)
C25A—C26A	1.398 (5)	C23C—C24C	1.397 (5)
C26A—C27A	1.395 (5)	C24C—C25C	1.384 (5)
C26A—C28A	1.522 (4)	C24C—H24C	0.9500
C27A—H27A	0.9500	C25C—C26C	1.397 (5)
C28A—C69A	1.543 (5)	C26C—C27C	1.401 (5)
C28A—H28A	1.0000	C26C—C28C	1.517 (4)
C29A—H29A	0.9800	C27C—H27C	0.9500
C29A—H29B	0.9800	C28C—C69C	1.548 (5)
C29A—H29C	0.9800	C28C—H28C	1.0000
C30A—C31A	1.500 (6)	C29C—H29G	0.9800
C30A—H30A	0.9900	C29C—H29H	0.9800
C30A—H30B	0.9900	C29C—H29I	0.9800
C32A—H32A	0.9800	C30C—C31C	1.521 (6)
C32A—H32B	0.9800	C30C—H30E	0.9900
C32A—H32C	0.9800	C30C—H30F	0.9900
C33A—C34A	1.535 (5)	C32C—H32G	0.9800
C33A—H33A	0.9900	C32C—H32H	0.9800
C33A—H33B	0.9900	C32C—H32I	0.9800
C34A—C35A	1.508 (5)	C33C—C34C	1.537 (5)
C34A—H34A	0.9900	C33C—H33E	0.9900
C34A—H34B	0.9900	C33C—H33F	0.9900
C35A—C40A	1.387 (5)	C34C—C35C	1.521 (5)
C35A—C36A	1.397 (5)	C34C—H34E	0.9900
C36A—C37A	1.378 (6)	C34C—H34F	0.9900
C36A—H36A	0.9500	C35C—C40C	1.370 (5)
C37A—C38A	1.380 (6)	C35C—C36C	1.387 (6)
C37A—H37A	0.9500	C36C—C37C	1.389 (7)
C38A—C39A	1.370 (6)	C36C—H36C	0.9500
C38A—H38A	0.9500	C37C—C38C	1.360 (7)
C39A—C40A	1.391 (6)	C37C—H37C	0.9500
C39A—H39A	0.9500	C38C—C39C	1.373 (7)
C40A—H40A	0.9500	C38C—H38C	0.9500
C41A—H41A	0.9800	C39C—C40C	1.395 (6)
C41A—H41B	0.9800	C39C—H39C	0.9500
C41A—H41C	0.9800	C40C—H40C	0.9500
C42A—C43A	1.513 (5)	C41C—H41G	0.9800
C42A—H42A	0.9900	C41C—H41H	0.9800
C42A—H42B	0.9900	C41C—H41I	0.9800
C44A—H44A	0.9800	C42C—C43C	1.508 (6)
C44A—H44B	0.9800	C42C—H42E	0.9900
C44A—H44C	0.9800	C42C—H42F	0.9900
C45A—C46A	1.534 (5)	C44C—H44G	0.9800
C45A—H45A	0.9900	C44C—H44H	0.9800
C45A—H45B	0.9900	C44C—H44I	0.9800
C46A—C47A	1.500 (6)	C45C—C46C	1.524 (5)
C46A—H46A	0.9900	C45C—H45E	0.9900
C46A—H46B	0.9900	C45C—H45F	0.9900
C47A—C48A	1.390 (7)	C46C—C47C	1.521 (6)
C47A—C52A	1.393 (7)	C46C—H46E	0.9900
C48A—C49A	1.388 (7)	C46C—H46F	0.9900
C48A—H48A	0.9500	C47C—C52C	1.374 (7)
C49A—C50A	1.365 (8)	C47C—C48C	1.381 (7)
C49A—H49A	0.9500	C48C—C49C	1.377 (7)
C50A—C51A	1.372 (8)	C48C—H48C	0.9500
C50A—H50A	0.9500	C49C—C50C	1.377 (8)
C51A—C52A	1.372 (7)	C49C—H49C	0.9500
C51A—H51A	0.9500	C50C—C51C	1.357 (8)
C52A—H52A	0.9500	C50C—H50C	0.9500
C53A—H53A	0.9800	C51C—C52C	1.390 (7)
C53A—H53B	0.9800	C51C—H51C	0.9500
C53A—H53C	0.9800	C52C—H52C	0.9500
C54A—C55A	1.503 (6)	C53C—H53G	0.9800
C54A—H54A	0.9900	C53C—H53H	0.9800
C54A—H54B	0.9900	C53C—H53I	0.9800
C56A—H56A	0.9800	C56C—H56G	0.9800
C56A—H56B	0.9800	C56C—H56H	0.9800
C56A—H56C	0.9800	C56C—H56I	0.9800
C57A—C58A	1.532 (5)	C57C—C58C	1.527 (5)
C57A—H57A	0.9900	C57C—H57E	0.9900
C57A—H57B	0.9900	C57C—H57F	0.9900
C58A—C59A	1.496 (6)	C58C—C59C	1.508 (6)
C58A—H58A	0.9900	C58C—H58E	0.9900
C58A—H58B	0.9900	C58C—H58F	0.9900
C59A—C60A	1.373 (7)	C59C—C60C	1.368 (7)
C59A—C64A	1.398 (6)	C59C—C64C	1.392 (6)
C60A—C61A	1.406 (6)	C60C—C61C	1.387 (7)
C60A—H60A	0.9500	C60C—H60C	0.9500
C61A—C62A	1.354 (8)	C61C—C62C	1.370 (9)
C61A—H61A	0.9500	C61C—H61C	0.9500
C62A—C63A	1.360 (9)	C62C—C63C	1.373 (10)
C62A—H62A	0.9500	C62C—H62C	0.9500
C63A—C64A	1.397 (8)	C63C—C64C	1.371 (9)
C63A—H63A	0.9500	C63C—H63C	0.9500
C64A—H64A	0.9500	C64C—H64C	0.9500
C65A—H65A	0.9800	C65C—H65G	0.9800
C65A—H65B	0.9800	C65C—H65H	0.9800
C65A—H65C	0.9800	C65C—H65I	0.9800
C66A—C67A	1.513 (6)	C66C—C67C	1.485 (6)
C66A—H66A	0.9900	C66C—H66E	0.9900
C66A—H66B	0.9900	C66C—H66F	0.9900
C68A—H68A	0.9800	C68C—H68G	0.9800
C68A—H68B	0.9800	C68C—H68H	0.9800
C68A—H68C	0.9800	C68C—H68I	0.9800
C69A—C70A	1.542 (5)	C69C—C70C	1.539 (5)
C69A—H69A	0.9900	C69C—H69E	0.9900
C69A—H69B	0.9900	C69C—H69F	0.9900
C70A—C71A	1.509 (5)	C70C—C71C	1.498 (6)
C70A—H70A	0.9900	C70C—H70E	0.9900
C70A—H70B	0.9900	C70C—H70F	0.9900
C71A—C76A	1.376 (6)	C71C—C72C	1.384 (7)
C71A—C72A	1.405 (6)	C71C—C76C	1.391 (7)
C72A—C73A	1.388 (6)	C72C—C73C	1.380 (8)
C72A—H72A	0.9500	C72C—H72C	0.9500
C73A—C74A	1.379 (7)	C73C—C74C	1.384 (13)
C73A—H73A	0.9500	C73C—H73C	0.9500
C74A—C75A	1.374 (8)	C74C—C75C	1.394 (13)
C74A—H74A	0.9500	C74C—H74C	0.9500
C75A—C76A	1.407 (7)	C75C—C76C	1.394 (9)
C75A—H75A	0.9500	C75C—H75C	0.9500
C76A—H76A	0.9500	C76C—H76C	0.9500
O1B—C2B	1.380 (4)	O1D—C2D	1.370 (4)
O1B—C29B	1.425 (4)	O1D—C29D	1.429 (4)
O2B—C4B	1.380 (4)	O2D—C4D	1.394 (4)
O2B—C30B	1.426 (4)	O2D—C30D	1.436 (4)
O3B—C31B	1.202 (5)	O3D—C31D	1.190 (5)
O4B—C31B	1.317 (5)	O4D—C31D	1.334 (6)
O4B—C32B	1.425 (7)	O4D—C32D	1.453 (6)
O5B—C9B	1.360 (4)	O5D—C9D	1.378 (4)
O5B—C41B	1.420 (5)	O5D—C41D	1.421 (5)
O6B—C11B	1.389 (4)	O6D—C11D	1.396 (4)
O6B—C42B	1.433 (5)	O6D—C42D	1.423 (4)
O7B—C43B	1.209 (7)	O7D—C43D	1.203 (5)
O8B—C43B	1.303 (7)	O8D—C43D	1.325 (5)
O8B—C44B	1.461 (6)	O8D—C44D	1.448 (5)
O9B—C16B	1.375 (4)	O9D—C16D	1.372 (5)
O9B—C53B	1.414 (5)	O9D—C53D	1.403 (5)
O10B—C18B	1.397 (4)	O10D—C18D	1.384 (5)
O10B—C54B	1.420 (5)	O10D—C54D	1.409 (5)
O11B—C55B	1.178 (6)	O11D—C55D	1.176 (6)
O12B—C55B	1.346 (7)	O12D—C55D	1.326 (7)
O12B—C56B	1.444 (8)	O12D—C56D	1.466 (6)
O13B—C23B	1.371 (4)	O13D—C23D	1.378 (4)
O13B—C65B	1.415 (5)	O13D—C65D	1.420 (5)
O14B—C25B	1.398 (4)	O14D—C25D	1.378 (4)
O14B—C66B	1.416 (5)	O14D—C66D	1.402 (4)
O15B—C67B	1.190 (5)	O15D—C67D	1.212 (5)
O16B—C67B	1.316 (5)	O16D—C67D	1.304 (5)
O16B—C68B	1.468 (5)	O16D—C68D	1.459 (4)
C1B—C6B	1.386 (5)	C1D—C6D	1.383 (5)
C1B—C2B	1.394 (4)	C1D—C2D	1.416 (4)
C1B—C28B	1.528 (5)	C1D—C28D	1.519 (5)
C2B—C3B	1.392 (5)	C2D—C3D	1.379 (5)
C3B—C4B	1.396 (5)	C3D—C4D	1.393 (5)
C3B—H3B	0.9500	C3D—H3D	0.9500
C4B—C5B	1.401 (4)	C4D—C5D	1.398 (4)
C5B—C6B	1.387 (5)	C5D—C6D	1.395 (5)
C5B—C7B	1.519 (5)	C5D—C7D	1.521 (5)
C6B—H6B	0.9500	C6D—H6D	0.9500
C7B—C8B	1.528 (4)	C7D—C8D	1.530 (4)
C7B—C33B	1.537 (5)	C7D—C33D	1.542 (5)
C7B—H7B	1.0000	C7D—H7D	1.0000
C8B—C13B	1.383 (4)	C8D—C9D	1.393 (5)
C8B—C9B	1.406 (5)	C8D—C13D	1.399 (4)
C9B—C10B	1.385 (5)	C9D—C10D	1.388 (5)
C10B—C11B	1.390 (5)	C10D—C11D	1.387 (5)
C10B—H10B	0.9500	C10D—H10D	0.9500
C11B—C12B	1.391 (5)	C11D—C12D	1.381 (5)
C12B—C13B	1.401 (5)	C12D—C13D	1.394 (4)
C12B—C14B	1.517 (4)	C12D—C14D	1.533 (4)
C13B—H13B	0.9500	C13D—H13D	0.9500
C14B—C15B	1.527 (5)	C14D—C15D	1.516 (5)
C14B—C45B	1.535 (5)	C14D—C45D	1.533 (5)
C14B—H14B	1.0000	C14D—H14D	1.0000
C15B—C20B	1.381 (5)	C15D—C20D	1.386 (5)
C15B—C16B	1.402 (4)	C15D—C16D	1.404 (5)
C16B—C17B	1.387 (5)	C16D—C17D	1.388 (6)
C17B—C18B	1.394 (5)	C17D—C18D	1.398 (6)
C17B—H17B	0.9500	C17D—H17D	0.9500
C18B—C19B	1.396 (4)	C18D—C19D	1.387 (5)
C19B—C20B	1.398 (5)	C19D—C20D	1.386 (5)
C19B—C21B	1.525 (5)	C19D—C21D	1.530 (5)
C20B—H20B	0.9500	C20D—H20D	0.9500
C21B—C22B	1.521 (4)	C21D—C22D	1.528 (4)
C21B—C57B	1.544 (5)	C21D—C57D	1.546 (5)
C21B—H21B	1.0000	C21D—H21D	1.0000
C22B—C27B	1.396 (4)	C22D—C27D	1.392 (5)
C22B—C23B	1.408 (5)	C22D—C23D	1.394 (5)
C23B—C24B	1.383 (5)	C23D—C24D	1.393 (5)
C24B—C25B	1.383 (5)	C24D—C25D	1.389 (5)
C24B—H24B	0.9500	C24D—H24D	0.9500
C25B—C26B	1.393 (5)	C25D—C26D	1.394 (5)
C26B—C27B	1.391 (4)	C26D—C27D	1.394 (4)
C26B—C28B	1.524 (4)	C26D—C28D	1.530 (4)
C27B—H27B	0.9500	C27D—H27D	0.9500
C28B—C69B	1.527 (5)	C28D—C69D	1.536 (5)
C28B—H28B	1.0000	C28D—H28D	1.0000
C29B—H29D	0.9800	C29D—H29J	0.9800
C29B—H29E	0.9800	C29D—H29K	0.9800
C29B—H29F	0.9800	C29D—H29L	0.9800
C30B—C31B	1.483 (6)	C30D—C31D	1.499 (5)
C30B—H30C	0.9900	C30D—H30G	0.9900
C30B—H30D	0.9900	C30D—H30H	0.9900
C32B—H32D	0.9800	C32D—H32J	0.9800
C32B—H32E	0.9800	C32D—H32K	0.9800
C32B—H32F	0.9800	C32D—H32L	0.9800
C33B—C34B	1.539 (5)	C33D—C34D	1.535 (5)
C33B—H33C	0.9900	C33D—H33G	0.9900
C33B—H33D	0.9900	C33D—H33H	0.9900
C34B—C35B	1.514 (5)	C34D—C35D	1.511 (5)
C34B—H34C	0.9900	C34D—H34G	0.9900
C34B—H34D	0.9900	C34D—H34H	0.9900
C35B—C36B	1.370 (6)	C35D—C40D	1.388 (5)
C35B—C40B	1.387 (6)	C35D—C36D	1.398 (5)
C36B—C37B	1.385 (6)	C36D—C37D	1.402 (6)
C36B—H36B	0.9500	C36D—H36D	0.9500
C37B—C38B	1.377 (6)	C37D—C38D	1.368 (7)
C37B—H37B	0.9500	C37D—H37D	0.9500
C38B—C39B	1.374 (7)	C38D—C39D	1.376 (7)
C38B—H38B	0.9500	C38D—H38D	0.9500
C39B—C40B	1.377 (6)	C39D—C40D	1.395 (6)
C39B—H39B	0.9500	C39D—H39D	0.9500
C40B—H40B	0.9500	C40D—H40D	0.9500
C41B—H41D	0.9800	C41D—H41J	0.9800
C41B—H41E	0.9800	C41D—H41K	0.9800
C41B—H41F	0.9800	C41D—H41L	0.9800
C42B—C43B	1.491 (6)	C42D—C43D	1.511 (5)
C42B—H42C	0.9900	C42D—H42G	0.9900
C42B—H42D	0.9900	C42D—H42H	0.9900
C44B—H44D	0.9800	C44D—H44J	0.9800
C44B—H44E	0.9800	C44D—H44K	0.9800
C44B—H44F	0.9800	C44D—H44L	0.9800
C45B—C46B	1.538 (5)	C45D—C46D	1.531 (5)
C45B—H45C	0.9900	C45D—H45G	0.9900
C45B—H45D	0.9900	C45D—H45H	0.9900
C46B—C47B	1.519 (5)	C46D—C47D	1.509 (6)
C46B—H46C	0.9900	C46D—H46G	0.9900
C46B—H46D	0.9900	C46D—H46H	0.9900
C47B—C52B	1.378 (6)	C47D—C48D	1.376 (7)
C47B—C48B	1.384 (6)	C47D—C52D	1.390 (7)
C48B—C49B	1.387 (7)	C48D—C49D	1.384 (7)
C48B—H48B	0.9500	C48D—H48D	0.9500
C49B—C50B	1.360 (8)	C49D—C50D	1.390 (8)
C49B—H49B	0.9500	C49D—H49D	0.9500
C50B—C51B	1.395 (7)	C50D—C51D	1.360 (8)
C50B—H50B	0.9500	C50D—H50D	0.9500
C51B—C52B	1.390 (7)	C51D—C52D	1.391 (7)
C51B—H51B	0.9500	C51D—H51D	0.9500
C52B—H52B	0.9500	C52D—H52D	0.9500
C53B—H53D	0.9800	C53D—H53J	0.9800
C53B—H53E	0.9800	C53D—H53K	0.9800
C53B—H53F	0.9800	C53D—H53L	0.9800
C54B—C55B	1.520 (6)	C54D—C55D	1.494 (7)
C54B—H54C	0.9900	C54D—H54G	0.9900
C54B—H54D	0.9900	C54D—H54H	0.9900
C56B—H56D	0.9800	C56D—H56J	0.9800
C56B—H56E	0.9800	C56D—H56K	0.9800
C56B—H56F	0.9800	C56D—H56L	0.9800
C57B—C58B	1.536 (5)	C57D—C58D	1.527 (5)
C57B—H57C	0.9900	C57D—H57G	0.9900
C57B—H57D	0.9900	C57D—H57H	0.9900
C58B—C59B	1.507 (5)	C58D—C59D	1.510 (6)
C58B—H58C	0.9900	C58D—H58G	0.9900
C58B—H58D	0.9900	C58D—H58H	0.9900
C59B—C64B	1.386 (5)	C59D—C64D	1.368 (7)
C59B—C60B	1.388 (5)	C59D—C60D	1.369 (7)
C60B—C61B	1.385 (6)	C60D—C61D	1.392 (8)
C60B—H60B	0.9500	C60D—H60D	0.9500
C61B—C62B	1.379 (6)	C61D—C62D	1.368 (9)
C61B—H61B	0.9500	C61D—H61D	0.9500
C62B—C63B	1.363 (6)	C62D—C63D	1.348 (9)
C62B—H62B	0.9500	C62D—H62D	0.9500
C63B—C64B	1.390 (6)	C63D—C64D	1.399 (8)
C63B—H63B	0.9500	C63D—H63D	0.9500
C64B—H64B	0.9500	C64D—H64D	0.9500
C65B—H65D	0.9800	C65D—H65J	0.9800
C65B—H65E	0.9800	C65D—H65K	0.9800
C65B—H65F	0.9800	C65D—H65L	0.9800
C66B—C67B	1.527 (5)	C66D—C67D	1.513 (5)
C66B—H66C	0.9900	C66D—H66G	0.9900
C66B—H66D	0.9900	C66D—H66H	0.9900
C68B—H68D	0.9800	C68D—H68J	0.9800
C68B—H68E	0.9800	C68D—H68K	0.9800
C68B—H68F	0.9800	C68D—H68L	0.9800
C69B—C70B	1.532 (5)	C69D—C70D	1.533 (5)
C69B—H69C	0.9900	C69D—H69G	0.9900
C69B—H69D	0.9900	C69D—H69H	0.9900
C70B—C71B	1.507 (6)	C70D—C71D	1.507 (6)
C70B—H70C	0.9900	C70D—H70G	0.9900
C70B—H70D	0.9900	C70D—H70H	0.9900
C71B—C76B	1.363 (7)	C71D—C72D	1.379 (7)
C71B—C72B	1.392 (7)	C71D—C76D	1.392 (8)
C72B—C73B	1.385 (7)	C72D—C73D	1.431 (11)
C72B—H72B	0.9500	C72D—H72D	0.9500
C73B—C74B	1.351 (9)	C73D—C74D	1.389 (13)
C73B—H73B	0.9500	C73D—H73D	0.9500
C74B—C75B	1.355 (9)	C74D—C75D	1.369 (13)
C74B—H74B	0.9500	C74D—H74D	0.9500
C75B—C76B	1.413 (8)	C75D—C76D	1.377 (8)
C75B—H75B	0.9500	C75D—H75D	0.9500
C76B—H76B	0.9500	C76D—H76D	0.9500
			
C29A—O1A—C2A—C3A	9.2 (6)	C29C—O1C—C2C—C3C	5.1 (6)
C29A—O1A—C2A—C1A	−169.7 (4)	C29C—O1C—C2C—C1C	−175.9 (4)
C6A—C1A—C2A—O1A	175.3 (3)	C6C—C1C—C2C—C3C	0.8 (5)
C28A—C1A—C2A—O1A	−1.1 (5)	C28C—C1C—C2C—C3C	176.5 (3)
C6A—C1A—C2A—C3A	−3.6 (5)	C6C—C1C—C2C—O1C	−178.3 (3)
C28A—C1A—C2A—C3A	−180.0 (3)	C28C—C1C—C2C—O1C	−2.5 (5)
O1A—C2A—C3A—C4A	−175.6 (3)	O1C—C2C—C3C—C4C	178.2 (3)
C1A—C2A—C3A—C4A	3.3 (5)	C1C—C2C—C3C—C4C	−0.8 (6)
C2A—C3A—C4A—C5A	−0.1 (5)	C2C—C3C—C4C—O2C	−178.0 (3)
C2A—C3A—C4A—O2A	176.5 (3)	C2C—C3C—C4C—C5C	0.4 (6)
C30A—O2A—C4A—C3A	102.1 (4)	C30C—O2C—C4C—C3C	−97.2 (4)
C30A—O2A—C4A—C5A	−81.3 (4)	C30C—O2C—C4C—C5C	84.4 (4)
C3A—C4A—C5A—C6A	−2.5 (5)	C3C—C4C—C5C—C6C	0.0 (5)
O2A—C4A—C5A—C6A	−179.0 (3)	O2C—C4C—C5C—C6C	178.4 (3)
C3A—C4A—C5A—C7A	171.2 (3)	C3C—C4C—C5C—C7C	−176.3 (3)
O2A—C4A—C5A—C7A	−5.3 (5)	O2C—C4C—C5C—C7C	2.0 (5)
C4A—C5A—C6A—C1A	2.1 (5)	C4C—C5C—C6C—C1C	0.0 (5)
C7A—C5A—C6A—C1A	−171.7 (3)	C7C—C5C—C6C—C1C	176.3 (3)
C2A—C1A—C6A—C5A	0.9 (5)	C2C—C1C—C6C—C5C	−0.3 (5)
C28A—C1A—C6A—C5A	177.3 (3)	C28C—C1C—C6C—C5C	−176.1 (3)
C6A—C5A—C7A—C8A	−43.1 (4)	C6C—C5C—C7C—C8C	41.1 (4)
C4A—C5A—C7A—C8A	143.5 (3)	C4C—C5C—C7C—C8C	−142.7 (3)
C6A—C5A—C7A—C33A	81.1 (4)	C6C—C5C—C7C—C33C	−85.9 (4)
C4A—C5A—C7A—C33A	−92.3 (4)	C4C—C5C—C7C—C33C	90.3 (4)
C5A—C7A—C8A—C13A	109.5 (4)	C33C—C7C—C8C—C13C	10.2 (5)
C33A—C7A—C8A—C13A	−10.8 (5)	C5C—C7C—C8C—C13C	−113.0 (3)
C5A—C7A—C8A—C9A	−71.8 (4)	C33C—C7C—C8C—C9C	−170.7 (3)
C33A—C7A—C8A—C9A	168.0 (3)	C5C—C7C—C8C—C9C	66.1 (4)
C41A—O5A—C9A—C10A	−3.7 (5)	C41C—O5C—C9C—C10C	3.2 (5)
C41A—O5A—C9A—C8A	177.4 (3)	C41C—O5C—C9C—C8C	−177.7 (3)
C13A—C8A—C9A—O5A	−178.5 (3)	C13C—C8C—C9C—O5C	178.8 (3)
C7A—C8A—C9A—O5A	2.7 (5)	C7C—C8C—C9C—O5C	−0.4 (5)
C13A—C8A—C9A—C10A	2.6 (5)	C13C—C8C—C9C—C10C	−2.1 (5)
C7A—C8A—C9A—C10A	−176.3 (3)	C7C—C8C—C9C—C10C	178.7 (3)
O5A—C9A—C10A—C11A	−178.7 (3)	O5C—C9C—C10C—C11C	179.2 (3)
C8A—C9A—C10A—C11A	0.2 (5)	C8C—C9C—C10C—C11C	0.2 (5)
C9A—C10A—C11A—C12A	−3.0 (5)	C42C—O6C—C11C—C10C	−67.8 (4)
C9A—C10A—C11A—O6A	172.7 (3)	C42C—O6C—C11C—C12C	116.2 (3)
C42A—O6A—C11A—C10A	73.0 (4)	C9C—C10C—C11C—O6C	−174.4 (3)
C42A—O6A—C11A—C12A	−111.2 (4)	C9C—C10C—C11C—C12C	1.4 (5)
C10A—C11A—C12A—C13A	2.7 (5)	O6C—C11C—C12C—C13C	175.0 (3)
O6A—C11A—C12A—C13A	−173.0 (3)	C10C—C11C—C12C—C13C	−1.0 (5)
C10A—C11A—C12A—C14A	−177.8 (3)	O6C—C11C—C12C—C14C	−5.7 (5)
O6A—C11A—C12A—C14A	6.5 (5)	C10C—C11C—C12C—C14C	178.3 (3)
C9A—C8A—C13A—C12A	−2.8 (5)	C9C—C8C—C13C—C12C	2.6 (5)
C7A—C8A—C13A—C12A	176.0 (3)	C7C—C8C—C13C—C12C	−178.3 (3)
C11A—C12A—C13A—C8A	0.2 (5)	C11C—C12C—C13C—C8C	−1.1 (5)
C14A—C12A—C13A—C8A	−179.2 (3)	C14C—C12C—C13C—C8C	179.7 (3)
C11A—C12A—C14A—C15A	69.7 (4)	C11C—C12C—C14C—C15C	−63.2 (4)
C13A—C12A—C14A—C15A	−110.9 (4)	C13C—C12C—C14C—C15C	116.1 (3)
C11A—C12A—C14A—C45A	−165.1 (3)	C11C—C12C—C14C—C45C	170.9 (3)
C13A—C12A—C14A—C45A	14.4 (5)	C13C—C12C—C14C—C45C	−9.8 (5)
C12A—C14A—C15A—C20A	50.4 (4)	C12C—C14C—C15C—C16C	129.2 (3)
C45A—C14A—C15A—C20A	−76.4 (4)	C45C—C14C—C15C—C16C	−103.6 (4)
C12A—C14A—C15A—C16A	−129.9 (3)	C12C—C14C—C15C—C20C	−46.6 (4)
C45A—C14A—C15A—C16A	103.3 (4)	C45C—C14C—C15C—C20C	80.5 (4)
C53A—O9A—C16A—C17A	9.7 (6)	C53C—O9C—C16C—C17C	−3.7 (6)
C53A—O9A—C16A—C15A	−171.4 (4)	C53C—O9C—C16C—C15C	176.8 (4)
C20A—C15A—C16A—O9A	−176.7 (3)	C20C—C15C—C16C—O9C	−178.9 (3)
C14A—C15A—C16A—O9A	3.6 (5)	C14C—C15C—C16C—O9C	5.1 (5)
C20A—C15A—C16A—C17A	2.3 (5)	C20C—C15C—C16C—C17C	1.7 (5)
C14A—C15A—C16A—C17A	−177.4 (3)	C14C—C15C—C16C—C17C	−174.3 (3)
O9A—C16A—C17A—C18A	178.3 (3)	O9C—C16C—C17C—C18C	−178.9 (4)
C15A—C16A—C17A—C18A	−0.5 (5)	C15C—C16C—C17C—C18C	0.5 (6)
C54A—O10A—C18A—C17A	1.5 (5)	C16C—C17C—C18C—O10C	178.1 (4)
C54A—O10A—C18A—C19A	−179.7 (3)	C16C—C17C—C18C—C19C	−3.6 (6)
C16A—C17A—C18A—O10A	176.7 (3)	O10C—C18C—C19C—C20C	−177.4 (3)
C16A—C17A—C18A—C19A	−1.9 (5)	C17C—C18C—C19C—C20C	4.2 (5)
O10A—C18A—C19A—C20A	−176.3 (3)	O10C—C18C—C19C—C21C	2.1 (5)
C17A—C18A—C19A—C20A	2.4 (5)	C17C—C18C—C19C—C21C	−176.4 (4)
O10A—C18A—C19A—C21A	4.7 (5)	C18C—C19C—C20C—C15C	−1.9 (5)
C17A—C18A—C19A—C21A	−176.5 (3)	C21C—C19C—C20C—C15C	178.6 (3)
C18A—C19A—C20A—C15A	−0.6 (5)	C16C—C15C—C20C—C19C	−1.0 (5)
C21A—C19A—C20A—C15A	178.4 (3)	C14C—C15C—C20C—C19C	175.1 (3)
C16A—C15A—C20A—C19A	−1.7 (5)	C20C—C19C—C21C—C22C	50.3 (5)
C14A—C15A—C20A—C19A	178.0 (3)	C18C—C19C—C21C—C22C	−129.2 (4)
C20A—C19A—C21A—C57A	83.0 (4)	C20C—C19C—C21C—C57C	−76.0 (4)
C18A—C19A—C21A—C57A	−98.1 (4)	C18C—C19C—C21C—C57C	104.6 (4)
C20A—C19A—C21A—C22A	−42.7 (4)	C19C—C21C—C22C—C27C	−111.7 (4)
C18A—C19A—C21A—C22A	136.2 (3)	C57C—C21C—C22C—C27C	13.3 (5)
C19A—C21A—C22A—C27A	113.5 (4)	C19C—C21C—C22C—C23C	69.6 (4)
C57A—C21A—C22A—C27A	−11.2 (5)	C57C—C21C—C22C—C23C	−165.4 (3)
C19A—C21A—C22A—C23A	−67.6 (4)	C65C—O13C—C23C—C24C	6.8 (5)
C57A—C21A—C22A—C23A	167.6 (3)	C65C—O13C—C23C—C22C	−173.6 (4)
C65A—O13A—C23A—C24A	−12.6 (5)	C27C—C22C—C23C—O13C	179.8 (3)
C65A—O13A—C23A—C22A	168.3 (3)	C21C—C22C—C23C—O13C	−1.4 (5)
C27A—C22A—C23A—O13A	178.9 (3)	C27C—C22C—C23C—C24C	−0.7 (5)
C21A—C22A—C23A—O13A	0.0 (5)	C21C—C22C—C23C—C24C	178.1 (3)
C27A—C22A—C23A—C24A	−0.1 (5)	O13C—C23C—C24C—C25C	179.8 (3)
C21A—C22A—C23A—C24A	−179.0 (3)	C22C—C23C—C24C—C25C	0.3 (5)
O13A—C23A—C24A—C25A	−178.0 (3)	C23C—C24C—C25C—C26C	0.2 (6)
C22A—C23A—C24A—C25A	1.0 (5)	C23C—C24C—C25C—O14C	−177.4 (3)
C66A—O14A—C25A—C24A	45.0 (5)	C66C—O14C—C25C—C24C	−58.6 (6)
C66A—O14A—C25A—C26A	−136.0 (4)	C66C—O14C—C25C—C26C	123.6 (5)
C23A—C24A—C25A—O14A	178.4 (3)	C24C—C25C—C26C—C27C	−0.3 (5)
C23A—C24A—C25A—C26A	−0.6 (5)	O14C—C25C—C26C—C27C	177.5 (3)
O14A—C25A—C26A—C27A	−179.7 (3)	C24C—C25C—C26C—C28C	177.9 (3)
C24A—C25A—C26A—C27A	−0.7 (5)	O14C—C25C—C26C—C28C	−4.4 (5)
O14A—C25A—C26A—C28A	3.2 (5)	C23C—C22C—C27C—C26C	0.6 (5)
C24A—C25A—C26A—C28A	−177.8 (3)	C21C—C22C—C27C—C26C	−178.2 (3)
C23A—C22A—C27A—C26A	−1.2 (5)	C25C—C26C—C27C—C22C	−0.1 (5)
C21A—C22A—C27A—C26A	177.7 (3)	C28C—C26C—C27C—C22C	−178.3 (3)
C25A—C26A—C27A—C22A	1.6 (5)	C6C—C1C—C28C—C26C	−46.2 (4)
C28A—C26A—C27A—C22A	178.6 (3)	C2C—C1C—C28C—C26C	138.2 (3)
C27A—C26A—C28A—C1A	−116.2 (4)	C6C—C1C—C28C—C69C	80.0 (4)
C25A—C26A—C28A—C1A	60.7 (4)	C2C—C1C—C28C—C69C	−95.6 (4)
C27A—C26A—C28A—C69A	7.2 (5)	C25C—C26C—C28C—C1C	−65.1 (4)
C25A—C26A—C28A—C69A	−176.0 (3)	C27C—C26C—C28C—C1C	113.0 (4)
C6A—C1A—C28A—C26A	41.3 (4)	C25C—C26C—C28C—C69C	173.0 (3)
C2A—C1A—C28A—C26A	−142.4 (3)	C27C—C26C—C28C—C69C	−8.9 (5)
C6A—C1A—C28A—C69A	−85.6 (4)	C4C—O2C—C30C—C31C	80.6 (4)
C2A—C1A—C28A—C69A	90.6 (4)	C32C—O4C—C31C—O3C	−0.3 (8)
C4A—O2A—C30A—C31A	−88.5 (4)	C32C—O4C—C31C—C30C	−178.8 (5)
C32A—O4A—C31A—O3A	2.8 (8)	O2C—C30C—C31C—O3C	23.6 (7)
C32A—O4A—C31A—C30A	−178.2 (5)	O2C—C30C—C31C—O4C	−158.0 (3)
O2A—C30A—C31A—O3A	−7.1 (7)	C8C—C7C—C33C—C34C	68.0 (4)
O2A—C30A—C31A—O4A	173.9 (3)	C5C—C7C—C33C—C34C	−167.3 (3)
C5A—C7A—C33A—C34A	168.7 (3)	C7C—C33C—C34C—C35C	−177.3 (3)
C8A—C7A—C33A—C34A	−67.9 (4)	C33C—C34C—C35C—C40C	89.3 (4)
C7A—C33A—C34A—C35A	174.2 (3)	C33C—C34C—C35C—C36C	−88.2 (5)
C33A—C34A—C35A—C40A	−97.4 (4)	C40C—C35C—C36C—C37C	0.0 (7)
C33A—C34A—C35A—C36A	80.2 (4)	C34C—C35C—C36C—C37C	177.5 (4)
C40A—C35A—C36A—C37A	0.8 (6)	C35C—C36C—C37C—C38C	0.4 (8)
C34A—C35A—C36A—C37A	−177.0 (4)	C36C—C37C—C38C—C39C	0.1 (8)
C35A—C36A—C37A—C38A	−0.9 (7)	C37C—C38C—C39C—C40C	−1.0 (8)
C36A—C37A—C38A—C39A	0.8 (7)	C36C—C35C—C40C—C39C	−0.8 (7)
C37A—C38A—C39A—C40A	−0.6 (7)	C34C—C35C—C40C—C39C	−178.4 (4)
C36A—C35A—C40A—C39A	−0.6 (6)	C38C—C39C—C40C—C35C	1.3 (7)
C34A—C35A—C40A—C39A	177.1 (4)	C11C—O6C—C42C—C43C	−176.8 (3)
C38A—C39A—C40A—C35A	0.5 (7)	C44C—O8C—C43C—O7C	1.3 (7)
C11A—O6A—C42A—C43A	173.9 (3)	C44C—O8C—C43C—C42C	−176.6 (4)
C44A—O8A—C43A—O7A	1.0 (6)	O6C—C42C—C43C—O7C	154.8 (4)
C44A—O8A—C43A—C42A	178.9 (4)	O6C—C42C—C43C—O8C	−27.2 (5)
O6A—C42A—C43A—O7A	−151.3 (4)	C15C—C14C—C45C—C46C	65.2 (4)
O6A—C42A—C43A—O8A	30.7 (5)	C12C—C14C—C45C—C46C	−170.0 (3)
C12A—C14A—C45A—C46A	170.6 (3)	C14C—C45C—C46C—C47C	170.9 (4)
C15A—C14A—C45A—C46A	−64.1 (4)	C45C—C46C—C47C—C52C	108.4 (4)
C14A—C45A—C46A—C47A	−171.9 (4)	C45C—C46C—C47C—C48C	−72.3 (5)
C45A—C46A—C47A—C48A	−109.1 (4)	C52C—C47C—C48C—C49C	−0.5 (7)
C45A—C46A—C47A—C52A	72.6 (5)	C46C—C47C—C48C—C49C	−179.8 (4)
C52A—C47A—C48A—C49A	0.8 (5)	C47C—C48C—C49C—C50C	2.4 (8)
C46A—C47A—C48A—C49A	−177.6 (4)	C48C—C49C—C50C—C51C	−2.4 (8)
C47A—C48A—C49A—C50A	−1.1 (6)	C49C—C50C—C51C—C52C	0.6 (7)
C48A—C49A—C50A—C51A	0.8 (7)	C48C—C47C—C52C—C51C	−1.4 (6)
C49A—C50A—C51A—C52A	−0.2 (8)	C46C—C47C—C52C—C51C	177.9 (4)
C50A—C51A—C52A—C47A	−0.2 (8)	C50C—C51C—C52C—C47C	1.3 (6)
C48A—C47A—C52A—C51A	−0.1 (7)	C17C—C18C—O10C—C54C	−30.1 (6)
C46A—C47A—C52A—C51A	178.3 (4)	C19C—C18C—O10C—C54C	151.5 (4)
C18A—O10A—C54A—C55A	75.9 (4)	C55C—C54C—O10C—C18C	93.5 (6)
C56A—O12A—C55A—O11A	−0.9 (8)	C56C—O12C—C55C—O11C	0.4 (11)
C56A—O12A—C55A—C54A	175.6 (4)	C56C—O12C—C55C—C54C	−178.4 (6)
O10A—C54A—C55A—O11A	40.8 (7)	O10C—C54C—C55C—O11C	175.9 (7)
O10A—C54A—C55A—O12A	−135.7 (4)	O10C—C54C—C55C—O12C	−5.3 (8)
C19A—C21A—C57A—C58A	63.9 (4)	C19C—C21C—C57C—C58C	−60.4 (4)
C22A—C21A—C57A—C58A	−171.7 (3)	C22C—C21C—C57C—C58C	173.5 (3)
C21A—C57A—C58A—C59A	179.7 (4)	C21C—C57C—C58C—C59C	173.4 (3)
C57A—C58A—C59A—C60A	103.6 (4)	C57C—C58C—C59C—C60C	38.3 (6)
C57A—C58A—C59A—C64A	−73.5 (5)	C57C—C58C—C59C—C64C	−140.5 (4)
C64A—C59A—C60A—C61A	0.4 (6)	C64C—C59C—C60C—C61C	0.2 (8)
C58A—C59A—C60A—C61A	−176.9 (4)	C58C—C59C—C60C—C61C	−178.6 (5)
C59A—C60A—C61A—C62A	−1.5 (7)	C59C—C60C—C61C—C62C	0.3 (9)
C60A—C61A—C62A—C63A	0.8 (8)	C60C—C61C—C62C—C63C	−1.8 (9)
C61A—C62A—C63A—C64A	0.9 (8)	C61C—C62C—C63C—C64C	2.8 (9)
C62A—C63A—C64A—C59A	−2.0 (7)	C62C—C63C—C64C—C59C	−2.3 (9)
C60A—C59A—C64A—C63A	1.3 (6)	C60C—C59C—C64C—C63C	0.8 (7)
C58A—C59A—C64A—C63A	178.6 (4)	C58C—C59C—C64C—C63C	179.6 (5)
C25A—O14A—C66A—C67A	163.3 (4)	C25C—O14C—C66C—C67C	−174.7 (5)
C68A—O16A—C67A—O15A	−2.2 (8)	C68C—O16C—C67C—O15C	−2.3 (9)
C68A—O16A—C67A—C66A	177.2 (4)	C68C—O16C—C67C—C66C	179.0 (5)
O14A—C66A—C67A—O15A	−165.1 (5)	O14C—C66C—C67C—O15C	178.2 (6)
O14A—C66A—C67A—O16A	15.5 (6)	O14C—C66C—C67C—O16C	−3.1 (8)
C26A—C28A—C69A—C70A	68.2 (4)	C1C—C28C—C69C—C70C	166.4 (3)
C1A—C28A—C69A—C70A	−167.4 (3)	C26C—C28C—C69C—C70C	−69.8 (4)
C28A—C69A—C70A—C71A	−175.5 (3)	C28C—C69C—C70C—C71C	−174.4 (3)
C69A—C70A—C71A—C76A	−90.9 (5)	C69C—C70C—C71C—C72C	−75.2 (5)
C69A—C70A—C71A—C72A	84.1 (4)	C69C—C70C—C71C—C76C	100.0 (5)
C76A—C71A—C72A—C73A	−0.9 (7)	C76C—C71C—C72C—C73C	0.3 (8)
C70A—C71A—C72A—C73A	−176.0 (4)	C70C—C71C—C72C—C73C	175.4 (5)
C71A—C72A—C73A—C74A	0.8 (7)	C71C—C72C—C73C—C74C	−1.6 (10)
C72A—C73A—C74A—C75A	−0.8 (8)	C72C—C73C—C74C—C75C	2.6 (12)
C73A—C74A—C75A—C76A	0.8 (8)	C73C—C74C—C75C—C76C	−2.2 (13)
C72A—C71A—C76A—C75A	0.9 (7)	C72C—C71C—C76C—C75C	0.1 (8)
C70A—C71A—C76A—C75A	176.0 (4)	C70C—C71C—C76C—C75C	−175.1 (5)
C74A—C75A—C76A—C71A	−0.9 (8)	C74C—C75C—C76C—C71C	0.9 (10)
C29B—O1B—C2B—C3B	−8.1 (5)	C29D—O1D—C2D—C3D	−7.2 (5)
C29B—O1B—C2B—C1B	173.4 (4)	C29D—O1D—C2D—C1D	173.6 (3)
C6B—C1B—C2B—O1B	176.2 (3)	C6D—C1D—C2D—O1D	179.4 (3)
C28B—C1B—C2B—O1B	−3.5 (5)	C28D—C1D—C2D—O1D	2.0 (5)
C6B—C1B—C2B—C3B	−2.3 (5)	C6D—C1D—C2D—C3D	0.1 (5)
C28B—C1B—C2B—C3B	178.0 (3)	C28D—C1D—C2D—C3D	−177.2 (3)
O1B—C2B—C3B—C4B	−178.1 (3)	O1D—C2D—C3D—C4D	−178.9 (3)
C1B—C2B—C3B—C4B	0.3 (5)	C1D—C2D—C3D—C4D	0.3 (5)
C30B—O2B—C4B—C3B	−6.1 (5)	C2D—C3D—C4D—O2D	178.4 (3)
C30B—O2B—C4B—C5B	174.1 (3)	C2D—C3D—C4D—C5D	−0.8 (5)
C2B—C3B—C4B—O2B	−177.5 (3)	C30D—O2D—C4D—C3D	95.8 (4)
C2B—C3B—C4B—C5B	2.3 (5)	C30D—O2D—C4D—C5D	−85.0 (4)
O2B—C4B—C5B—C6B	177.0 (3)	C3D—C4D—C5D—C6D	0.9 (5)
C3B—C4B—C5B—C6B	−2.9 (5)	O2D—C4D—C5D—C6D	−178.3 (3)
O2B—C4B—C5B—C7B	−5.2 (5)	C3D—C4D—C5D—C7D	176.4 (3)
C3B—C4B—C5B—C7B	175.0 (3)	O2D—C4D—C5D—C7D	−2.7 (5)
C2B—C1B—C6B—C5B	1.8 (5)	C2D—C1D—C6D—C5D	−0.1 (5)
C28B—C1B—C6B—C5B	−178.5 (3)	C28D—C1D—C6D—C5D	177.3 (3)
C4B—C5B—C6B—C1B	0.8 (5)	C4D—C5D—C6D—C1D	−0.4 (5)
C7B—C5B—C6B—C1B	−177.1 (3)	C7D—C5D—C6D—C1D	−176.0 (3)
C6B—C5B—C7B—C8B	43.9 (4)	C6D—C5D—C7D—C8D	−42.2 (4)
C4B—C5B—C7B—C8B	−133.9 (3)	C4D—C5D—C7D—C8D	142.5 (3)
C6B—C5B—C7B—C33B	−83.0 (4)	C6D—C5D—C7D—C33D	84.7 (4)
C4B—C5B—C7B—C33B	99.2 (4)	C4D—C5D—C7D—C33D	−90.6 (4)
C5B—C7B—C8B—C13B	−111.6 (4)	C5D—C7D—C8D—C9D	−67.1 (4)
C33B—C7B—C8B—C13B	14.9 (4)	C33D—C7D—C8D—C9D	169.0 (3)
C5B—C7B—C8B—C9B	68.6 (4)	C5D—C7D—C8D—C13D	113.6 (3)
C33B—C7B—C8B—C9B	−164.9 (3)	C33D—C7D—C8D—C13D	−10.3 (4)
C41B—O5B—C9B—C10B	9.4 (6)	C41D—O5D—C9D—C10D	−8.7 (5)
C41B—O5B—C9B—C8B	−170.6 (4)	C41D—O5D—C9D—C8D	172.1 (3)
C13B—C8B—C9B—O5B	179.5 (3)	C13D—C8D—C9D—O5D	−178.9 (3)
C7B—C8B—C9B—O5B	−0.7 (5)	C7D—C8D—C9D—O5D	1.8 (4)
C13B—C8B—C9B—C10B	−0.4 (5)	C13D—C8D—C9D—C10D	1.9 (5)
C7B—C8B—C9B—C10B	179.4 (3)	C7D—C8D—C9D—C10D	−177.4 (3)
O5B—C9B—C10B—C11B	179.9 (4)	O5D—C9D—C10D—C11D	−179.3 (3)
C8B—C9B—C10B—C11B	−0.2 (6)	C8D—C9D—C10D—C11D	−0.1 (5)
C42B—O6B—C11B—C10B	−64.0 (5)	C9D—C10D—C11D—C12D	−1.2 (5)
C42B—O6B—C11B—C12B	117.4 (4)	C9D—C10D—C11D—O6D	175.3 (3)
C9B—C10B—C11B—O6B	−178.2 (3)	C42D—O6D—C11D—C12D	−117.5 (3)
C9B—C10B—C11B—C12B	0.3 (6)	C42D—O6D—C11D—C10D	66.0 (4)
O6B—C11B—C12B—C13B	178.8 (3)	C10D—C11D—C12D—C13D	0.6 (5)
C10B—C11B—C12B—C13B	0.3 (5)	O6D—C11D—C12D—C13D	−175.8 (3)
O6B—C11B—C12B—C14B	−1.8 (5)	C10D—C11D—C12D—C14D	−177.5 (3)
C10B—C11B—C12B—C14B	179.6 (3)	O6D—C11D—C12D—C14D	6.0 (5)
C9B—C8B—C13B—C12B	1.1 (5)	C11D—C12D—C13D—C8D	1.2 (5)
C7B—C8B—C13B—C12B	−178.7 (3)	C14D—C12D—C13D—C8D	179.3 (3)
C11B—C12B—C13B—C8B	−1.0 (5)	C9D—C8D—C13D—C12D	−2.4 (5)
C14B—C12B—C13B—C8B	179.7 (3)	C7D—C8D—C13D—C12D	176.9 (3)
C11B—C12B—C14B—C15B	−64.3 (4)	C11D—C12D—C14D—C15D	62.6 (4)
C13B—C12B—C14B—C15B	115.0 (4)	C13D—C12D—C14D—C15D	−115.4 (4)
C11B—C12B—C14B—C45B	173.1 (3)	C11D—C12D—C14D—C45D	−170.4 (3)
C13B—C12B—C14B—C45B	−7.6 (5)	C13D—C12D—C14D—C45D	11.6 (5)
C12B—C14B—C15B—C20B	−41.6 (4)	C45D—C14D—C15D—C20D	−79.5 (4)
C45B—C14B—C15B—C20B	85.3 (4)	C12D—C14D—C15D—C20D	47.5 (4)
C12B—C14B—C15B—C16B	143.4 (3)	C45D—C14D—C15D—C16D	105.2 (4)
C45B—C14B—C15B—C16B	−89.7 (4)	C12D—C14D—C15D—C16D	−127.7 (3)
C53B—O9B—C16B—C17B	−14.0 (7)	C53D—O9D—C16D—C17D	1.4 (6)
C53B—O9B—C16B—C15B	165.5 (5)	C53D—O9D—C16D—C15D	−179.8 (4)
C20B—C15B—C16B—O9B	−176.4 (3)	C20D—C15D—C16D—O9D	178.9 (3)
C14B—C15B—C16B—O9B	−1.3 (5)	C14D—C15D—C16D—O9D	−5.7 (5)
C20B—C15B—C16B—C17B	3.1 (5)	C20D—C15D—C16D—C17D	−2.3 (5)
C14B—C15B—C16B—C17B	178.3 (3)	C14D—C15D—C16D—C17D	173.2 (3)
O9B—C16B—C17B—C18B	177.3 (4)	O9D—C16D—C17D—C18D	178.9 (4)
C15B—C16B—C17B—C18B	−2.2 (6)	C15D—C16D—C17D—C18D	0.1 (6)
C16B—C17B—C18B—C19B	−0.7 (6)	C54D—O10D—C18D—C19D	−151.7 (4)
C16B—C17B—C18B—O10B	−176.4 (3)	C54D—O10D—C18D—C17D	28.7 (6)
C54B—O10B—C18B—C17B	−102.2 (4)	C16D—C17D—C18D—O10D	−178.0 (4)
C54B—O10B—C18B—C19B	82.0 (4)	C16D—C17D—C18D—C19D	2.3 (6)
C17B—C18B—C19B—C20B	2.5 (5)	O10D—C18D—C19D—C20D	178.0 (3)
O10B—C18B—C19B—C20B	178.2 (3)	C17D—C18D—C19D—C20D	−2.4 (5)
C17B—C18B—C19B—C21B	−171.3 (3)	O10D—C18D—C19D—C21D	−3.3 (5)
O10B—C18B—C19B—C21B	4.4 (5)	C17D—C18D—C19D—C21D	176.4 (3)
C16B—C15B—C20B—C19B	−1.2 (5)	C18D—C19D—C20D—C15D	0.0 (5)
C14B—C15B—C20B—C19B	−176.4 (3)	C21D—C19D—C20D—C15D	−178.7 (3)
C18B—C19B—C20B—C15B	−1.5 (5)	C16D—C15D—C20D—C19D	2.2 (5)
C21B—C19B—C20B—C15B	172.4 (3)	C14D—C15D—C20D—C19D	−173.2 (3)
C18B—C19B—C21B—C22B	−142.4 (3)	C20D—C19D—C21D—C22D	−50.8 (4)
C20B—C19B—C21B—C22B	44.1 (4)	C18D—C19D—C21D—C22D	130.5 (3)
C18B—C19B—C21B—C57B	91.3 (4)	C20D—C19D—C21D—C57D	75.9 (4)
C20B—C19B—C21B—C57B	−82.2 (4)	C18D—C19D—C21D—C57D	−102.8 (4)
C19B—C21B—C22B—C27B	−110.1 (3)	C19D—C21D—C22D—C27D	114.1 (4)
C57B—C21B—C22B—C27B	12.5 (4)	C57D—C21D—C22D—C27D	−11.0 (5)
C19B—C21B—C22B—C23B	70.0 (4)	C19D—C21D—C22D—C23D	−66.7 (4)
C57B—C21B—C22B—C23B	−167.4 (3)	C57D—C21D—C22D—C23D	168.3 (3)
C65B—O13B—C23B—C24B	2.9 (5)	C65D—O13D—C23D—C24D	−9.9 (5)
C65B—O13B—C23B—C22B	−179.4 (3)	C65D—O13D—C23D—C22D	171.0 (3)
C27B—C22B—C23B—O13B	178.4 (3)	C27D—C22D—C23D—O13D	−177.7 (3)
C21B—C22B—C23B—O13B	−1.7 (5)	C21D—C22D—C23D—O13D	2.9 (5)
C27B—C22B—C23B—C24B	−3.8 (5)	C27D—C22D—C23D—C24D	3.2 (5)
C21B—C22B—C23B—C24B	176.1 (3)	C21D—C22D—C23D—C24D	−176.1 (3)
O13B—C23B—C24B—C25B	179.2 (3)	O13D—C23D—C24D—C25D	179.9 (3)
C22B—C23B—C24B—C25B	1.6 (5)	C22D—C23D—C24D—C25D	−1.1 (5)
C23B—C24B—C25B—C26B	1.2 (5)	C66D—O14D—C25D—C24D	15.7 (5)
C23B—C24B—C25B—O14B	−172.0 (3)	C66D—O14D—C25D—C26D	−163.0 (3)
C66B—O14B—C25B—C24B	−75.8 (4)	C23D—C24D—C25D—O14D	179.2 (3)
C66B—O14B—C25B—C26B	110.8 (4)	C23D—C24D—C25D—C26D	−2.2 (5)
C24B—C25B—C26B—C27B	−1.5 (5)	O14D—C25D—C26D—C27D	−178.3 (3)
O14B—C25B—C26B—C27B	171.9 (3)	C24D—C25D—C26D—C27D	3.1 (5)
C24B—C25B—C26B—C28B	179.0 (3)	O14D—C25D—C26D—C28D	4.3 (5)
O14B—C25B—C26B—C28B	−7.7 (5)	C24D—C25D—C26D—C28D	−174.3 (3)
C25B—C26B—C27B—C22B	−1.0 (5)	C23D—C22D—C27D—C26D	−2.3 (5)
C28B—C26B—C27B—C22B	178.5 (3)	C21D—C22D—C27D—C26D	177.0 (3)
C23B—C22B—C27B—C26B	3.6 (5)	C25D—C26D—C27D—C22D	−0.8 (5)
C21B—C22B—C27B—C26B	−176.3 (3)	C28D—C26D—C27D—C22D	176.5 (3)
C27B—C26B—C28B—C69B	−16.6 (5)	C6D—C1D—C28D—C26D	46.8 (4)
C25B—C26B—C28B—C69B	162.9 (3)	C2D—C1D—C28D—C26D	−136.0 (3)
C27B—C26B—C28B—C1B	109.8 (4)	C6D—C1D—C28D—C69D	−78.9 (4)
C25B—C26B—C28B—C1B	−70.7 (4)	C2D—C1D—C28D—C69D	98.3 (4)
C6B—C1B—C28B—C26B	−50.6 (4)	C27D—C26D—C28D—C1D	−114.1 (4)
C2B—C1B—C28B—C26B	129.1 (3)	C25D—C26D—C28D—C1D	63.1 (4)
C6B—C1B—C28B—C69B	77.5 (4)	C27D—C26D—C28D—C69D	7.2 (5)
C2B—C1B—C28B—C69B	−102.8 (4)	C25D—C26D—C28D—C69D	−175.6 (3)
C4B—O2B—C30B—C31B	−69.8 (4)	C4D—O2D—C30D—C31D	−80.3 (4)
C32B—O4B—C31B—O3B	2.3 (8)	C32D—O4D—C31D—O3D	1.5 (7)
C32B—O4B—C31B—C30B	−177.1 (5)	C32D—O4D—C31D—C30D	178.8 (4)
O2B—C30B—C31B—O3B	−22.0 (6)	O2D—C30D—C31D—O3D	−28.2 (6)
O2B—C30B—C31B—O4B	157.4 (4)	O2D—C30D—C31D—O4D	154.6 (3)
C5B—C7B—C33B—C34B	−63.3 (4)	C5D—C7D—C33D—C34D	166.2 (3)
C8B—C7B—C33B—C34B	170.8 (3)	C8D—C7D—C33D—C34D	−68.6 (4)
C7B—C33B—C34B—C35B	177.5 (3)	C7D—C33D—C34D—C35D	176.3 (3)
C33B—C34B—C35B—C36B	67.9 (5)	C33D—C34D—C35D—C40D	−91.3 (4)
C33B—C34B—C35B—C40B	−111.1 (4)	C33D—C34D—C35D—C36D	86.7 (4)
C40B—C35B—C36B—C37B	−0.6 (7)	C40D—C35D—C36D—C37D	1.7 (6)
C34B—C35B—C36B—C37B	−179.7 (4)	C34D—C35D—C36D—C37D	−176.3 (4)
C35B—C36B—C37B—C38B	−0.3 (7)	C35D—C36D—C37D—C38D	−1.6 (7)
C36B—C37B—C38B—C39B	1.0 (7)	C36D—C37D—C38D—C39D	0.7 (7)
C37B—C38B—C39B—C40B	−0.8 (7)	C37D—C38D—C39D—C40D	0.2 (7)
C38B—C39B—C40B—C35B	−0.1 (7)	C36D—C35D—C40D—C39D	−0.9 (6)
C36B—C35B—C40B—C39B	0.8 (6)	C34D—C35D—C40D—C39D	177.1 (4)
C34B—C35B—C40B—C39B	179.9 (4)	C38D—C39D—C40D—C35D	0.0 (7)
C11B—O6B—C42B—C43B	−162.5 (4)	C11D—O6D—C42D—C43D	179.3 (3)
C44B—O8B—C43B—O7B	−4.1 (9)	C44D—O8D—C43D—O7D	−0.5 (7)
C44B—O8B—C43B—C42B	176.3 (6)	C44D—O8D—C43D—C42D	175.7 (4)
O6B—C42B—C43B—O7B	171.6 (6)	O6D—C42D—C43D—O7D	−158.3 (4)
O6B—C42B—C43B—O8B	−8.7 (7)	O6D—C42D—C43D—O8D	25.4 (5)
C12B—C14B—C45B—C46B	−68.3 (4)	C15D—C14D—C45D—C46D	−65.7 (4)
C15B—C14B—C45B—C46B	167.5 (3)	C12D—C14D—C45D—C46D	169.8 (3)
C14B—C45B—C46B—C47B	173.1 (3)	C14D—C45D—C46D—C47D	−168.0 (4)
C45B—C46B—C47B—C52B	−74.4 (4)	C45D—C46D—C47D—C48D	71.6 (5)
C45B—C46B—C47B—C48B	106.2 (4)	C45D—C46D—C47D—C52D	−110.0 (4)
C52B—C47B—C48B—C49B	0.9 (7)	C52D—C47D—C48D—C49D	−1.5 (6)
C46B—C47B—C48B—C49B	−179.7 (4)	C46D—C47D—C48D—C49D	177.1 (4)
C47B—C48B—C49B—C50B	−0.6 (8)	C47D—C48D—C49D—C50D	0.9 (8)
C48B—C49B—C50B—C51B	0.6 (8)	C48D—C49D—C50D—C51D	−0.9 (7)
C49B—C50B—C51B—C52B	−0.9 (8)	C49D—C50D—C51D—C52D	1.5 (7)
C48B—C47B—C52B—C51B	−1.2 (7)	C48D—C47D—C52D—C51D	2.1 (6)
C46B—C47B—C52B—C51B	179.4 (4)	C46D—C47D—C52D—C51D	−176.4 (4)
C50B—C51B—C52B—C47B	1.2 (7)	C50D—C51D—C52D—C47D	−2.1 (6)
C18B—O10B—C54B—C55B	88.1 (4)	C18D—O10D—C54D—C55D	−94.1 (5)
C56B—O12B—C55B—O11B	−1.5 (9)	C56D—O12D—C55D—O11D	−3.1 (10)
C56B—O12B—C55B—C54B	178.4 (6)	C56D—O12D—C55D—C54D	−178.8 (6)
O10B—C54B—C55B—O11B	5.8 (7)	O10D—C54D—C55D—O11D	−170.8 (6)
O10B—C54B—C55B—O12B	−174.1 (4)	O10D—C54D—C55D—O12D	4.9 (7)
C22B—C21B—C57B—C58B	66.4 (4)	C22D—C21D—C57D—C58D	−166.4 (4)
C19B—C21B—C57B—C58B	−168.6 (3)	C19D—C21D—C57D—C58D	67.8 (4)
C21B—C57B—C58B—C59B	−173.8 (3)	C21D—C57D—C58D—C59D	−170.4 (4)
C57B—C58B—C59B—C64B	98.8 (4)	C57D—C58D—C59D—C64D	116.8 (5)
C57B—C58B—C59B—C60B	−77.1 (4)	C57D—C58D—C59D—C60D	−63.1 (6)
C64B—C59B—C60B—C61B	0.5 (6)	C64D—C59D—C60D—C61D	1.2 (9)
C58B—C59B—C60B—C61B	176.6 (4)	C58D—C59D—C60D—C61D	−178.9 (6)
C59B—C60B—C61B—C62B	0.0 (7)	C59D—C60D—C61D—C62D	−3.1 (11)
C60B—C61B—C62B—C63B	0.3 (7)	C60D—C61D—C62D—C63D	3.4 (11)
C61B—C62B—C63B—C64B	−1.1 (7)	C61D—C62D—C63D—C64D	−2.0 (10)
C60B—C59B—C64B—C63B	−1.4 (6)	C60D—C59D—C64D—C63D	0.2 (8)
C58B—C59B—C64B—C63B	−177.5 (4)	C58D—C59D—C64D—C63D	−179.7 (4)
C62B—C63B—C64B—C59B	1.7 (7)	C62D—C63D—C64D—C59D	0.3 (9)
C25B—O14B—C66B—C67B	−172.8 (3)	C25D—O14D—C66D—C67D	172.7 (3)
C68B—O16B—C67B—O15B	−1.6 (6)	C68D—O16D—C67D—O15D	−4.3 (7)
C68B—O16B—C67B—C66B	−179.6 (4)	C68D—O16D—C67D—C66D	170.9 (4)
O14B—C66B—C67B—O15B	150.2 (4)	O14D—C66D—C67D—O15D	−155.8 (5)
O14B—C66B—C67B—O16B	−31.8 (5)	O14D—C66D—C67D—O16D	28.9 (6)
C26B—C28B—C69B—C70B	−168.7 (3)	C1D—C28D—C69D—C70D	−167.6 (3)
C1B—C28B—C69B—C70B	64.8 (4)	C26D—C28D—C69D—C70D	70.5 (4)
C28B—C69B—C70B—C71B	172.6 (4)	C28D—C69D—C70D—C71D	178.8 (3)
C69B—C70B—C71B—C76B	104.1 (4)	C69D—C70D—C71D—C72D	−90.2 (5)
C69B—C70B—C71B—C72B	−76.8 (5)	C69D—C70D—C71D—C76D	84.1 (5)
C76B—C71B—C72B—C73B	0.6 (7)	C76D—C71D—C72D—C73D	−1.6 (8)
C70B—C71B—C72B—C73B	−178.5 (4)	C70D—C71D—C72D—C73D	172.8 (5)
C71B—C72B—C73B—C74B	0.5 (8)	C71D—C72D—C73D—C74D	2.0 (10)
C72B—C73B—C74B—C75B	−0.5 (9)	C72D—C73D—C74D—C75D	−1.0 (13)
C73B—C74B—C75B—C76B	−0.5 (8)	C73D—C74D—C75D—C76D	−0.5 (13)
C72B—C71B—C76B—C75B	−1.6 (6)	C74D—C75D—C76D—C71D	0.9 (10)
C70B—C71B—C76B—C75B	177.4 (4)	C72D—C71D—C76D—C75D	0.2 (8)
C74B—C75B—C76B—C71B	1.6 (7)	C70D—C71D—C76D—C75D	−174.3 (5)

### Hydrogen-bond geometry (Å, °)

**Table:**

*D*—H···*A*	*D*—H	H···*A*	*D*···*A*	*D*—H···*A*
C14B—H14B···O16A^i^	1.00	2.59	3.440 (4)	142
C30B—H30C···O11A^i^	0.99	2.49	3.464 (6)	169
C30C—H30F···O8D^ii^	0.99	2.55	3.259 (4)	128
C30D—H30G···O8C^iii^	0.99	2.56	3.270 (5)	129
C44A—H44B···O10B^iv^	0.98	2.54	3.292 (5)	133
C56C—H56H···O7B^v^	0.98	2.55	3.375 (9)	141
C56D—H56K···O15A^vi^	0.98	2.37	3.251 (8)	149
C62C—H62C···O7C^vii^	0.95	2.46	3.313 (6)	149
C62D—H62D···O7D^vii^	0.95	2.59	3.421 (7)	146
C63A—H63A···O3B^viii^	0.95	2.37	3.267 (7)	156

Symmetry codes: (i) *x*, *y*+1, *z*; (ii) −*x*+1, *y*−1/2, −*z*+2; (iii) −*x*+1, *y*+1/2, −*z*+2; (iv) −*x*+2, *y*−1/2, −*z*+1; (v) −*x*+2, *y*−1/2, −*z*+2; (vi) −*x*+2, *y*+1/2, −*z*+2; (vii) *x*+1, *y*, *z*; (viii) *x*−1, *y*−1, *z*.
